# Don’t let it distract you: how information about the availability of reward affects attentional selection

**DOI:** 10.3758/s13414-017-1376-8

**Published:** 2017-07-21

**Authors:** Michel Failing, Jan Theeuwes

**Affiliations:** Department of Experimental and Applied Psychology, Vrije Universiteit Amsterdam, 1081 BT Amsterdam, The Netherlands

**Keywords:** Reward, Attention, Attentional capture, Incentive motivation, Value-driven attentional capture

## Abstract

Previous research has shown that attentional selection is affected by reward contingencies: previously selected and rewarded stimuli continue to capture attention even if the reward contingencies are no longer in place. In the current study, we investigated whether attentional selection also is affected by stimuli that merely signal the magnitude of reward available on a given trial but, crucially, have never had instrumental value. In a series of experiments, we show that a stimulus signaling high reward availability captures attention even when that stimulus is and was never physically salient or part of the task set, and selecting it is harmful for obtaining reward. Our results suggest that irrelevant reward-signaling stimuli capture attention, because participants have learned about the relationship between the stimulus and reward. Importantly, we only observed learning after initial attentional prioritization of the reward signaling stimulus. We conclude that nonsalient, task-irrelevant but reward-signaling stimuli can affect attentional selection above and beyond top-down or bottom-up attentional control, however, only after such stimuli were initially prioritized for selection.

**Electronic supplementary material:**

The online version of this article (doi:10.3758/s13414-017-1376-8) contains supplementary material, which is available to authorized users.

## Introduction

Recently, there has been a surge of research into the sensitivity to different forms of reward (value) in humans. In particular, research on the interaction of reward and visual selection has rapidly grown in the last decade. Reward has been shown to have a substantial impact on visual processing by guiding attentional allocation, effectively modulating which stimuli are selected for further processing. This guidance is central to the visual system given its limited capacity for processing the vast amount of stimuli competing for attention (Theeuwes, 2010; Wolfe, 2007). Although many selection processes have been successfully explained by voluntary goal-directed (top-down) or involuntary stimulus-driven (bottom-up) processes, research has demonstrated that modulation in visual selection due to reward cannot be fully explained in terms of either one of those kind of processes (Awh, Belopolsky, & Theeuwes, 2012).

Key to the impact of reward on visual selection is its motivational component, which has long been thought to signal the prioritization of behavioral goals (Simon, 1967). More recently, a wealth of research has demonstrated that one way of achieving this prioritization is through fostering cognitive control, which allows for more efficient guidance of attention (Botvinick & Braver, 2015). For instance, reward in the form of monetary incentive cues moderates the resolution of perceptual conflict (Krebs, Boehler, Egner, & Woldorff, 2011) and improves both attentional filtering (Padmala & Pessoa, 2011) and target detection in visual tasks (Engelmann, Damaraju, Padmala, & Pessoa, 2009). Beneficial effects of incentive motivation also have been demonstrated in covert visual search and cueing tasks, in which the potential for earning relatively high reward boosts overall performance, evident in reduction of search time and/or increased accuracy for target discrimination (Kiss, Driver, & Eimer, 2009; Kristjansson, Sigurjonsdottir, & Driver, 2010). In such studies, reward available for adequate performance on a given task is signaled either at the start of a block (i.e., in a blocked design) or by being directly tied to a specific visual cue or feature of the target (Engelmann et al., 2009; Engelmann & Pessoa, 2007; Small, Gitelman, Simmons, Bloise, Parrish, & Mesulam, 2005; Sawaki, Luck, & Raymond, 2015). Importantly, reward in these tasks is congruent (i.e., not detrimental) to the task demands. For instance, the appearance of a reward signal before the onset of the search display may allow for optimal response preparation, while the appearance of a reward signal within the search display may improve task performance when the signal is tied to (i.e., not competing with) the spatial location or visual feature of the target. Thus, observing performance benefits under these circumstances is not surprising since it is strategically beneficial to integrate and prioritize the reward signal during selection so as to ensure a higher rate of success with the corollary of a larger reward payout.

It seems, however, not surprising that “tuning” cognitive control to reward comes at a cost when the reward signal is not tied to a task-relevant stimulus. For instance, studies have suggested that reward tied to a stimulus affects attentional selection even if they no longer predict reward. Importantly, such previously reward-associated stimuli interfered with target selection even when they were rendered completely irrelevant to the task at hand (Anderson, 2013; Chelazzi, Perlato, Santandrea, & Della Libera, 2013; Le Pelley, Mitchell, Beesley, George, & Wills, 2016). These studies typically consist of two separate phases (training and test). In the training phase, successful selection of the target is rewarded, resulting in a reward association for the specific visual feature of the target. Note that this training phase usually operates on the same premise as the incentive motivation studies noted in the previous paragraph, whereby the reward signal is congruent with the task demands. In the test phase, however, the reward signal becomes detrimental to the task demands: not only is reward no longer available, but the previously rewarded stimulus is also rendered a distractor that competes with a new target for selection. This approach is now well-documented in the literature on covert (Anderson, Laurent, & Yantis, 2011; Della Libera & Chelazzi, 2006, 2009; Failing & Theeuwes, 2014; Hickey, Chelazzi, & Theeuwes, 2010) as well as overt visual search (Anderson & Yantis, 2012; Bucker, Silvis, Donk, & Theeuwes, 2015; Theeuwes & Belopolsky, 2012). The vast majority of these studies converge onto similar findings: selection benefits for reward-associated stimuli during a training phase turn into behavioral costs (i.e., interference in search time) when these stimuli subsequently compete with a new target for selection, even if they are explicitly no longer predictive of reward.

One explanation for the persistent yet detrimental effect of selection bias toward reward signals is that reward has been associated with a particular “response,” such as an overt oculomotor or a covert attentional shift. This association carries over into a subsequent test phase in which it is no longer predictive of reward. Thus, after reward learning, the reoccurrence of a reward-associated stimulus elicits the learned (covert or overt) response even if that response no longer results in a reward. The automatically triggered response towards the reward-associated stimulus results in either performance benefits when the stimulus happens to be congruent with task demands (e.g., when it appears at or near the location of the target; Failing & Theeuwes, 2014; Kiss et al., 2009; Raymond & O’Brien, 2009) or performance costs when the stimulus happens to be incongruent with task demands (e.g., when it appears somewhere other than the target; Anderson et al., 2011; Failing & Theeuwes, 2014, 2015; Theeuwes & Belopolsky, 2012). As such, one can argue that these studies represent some form of instrumental conditioning in which a response to a stimulus feature that was predictive for reward is reenacted as soon as that conditioned stimulus reappears (Schultz, 2006).

In a recent study, Le Pelley et al. (2015) demonstrated attentional effects of reward that could not readily be explained in terms of instrumentally conditioning a response (see also Mine & Saiki, 2015). In one experiment, participants performed the additional singleton task (Theeuwes, 1992), searching for a shape singleton (e.g., a gray diamond) and reporting the orientation of a line segment inside of it. While some trials presented the target shape singleton among nontarget gray circles, other trials additionally presented a colored singleton distractor (e.g., a red or blue circle) that signaled the availability of reward for that particular trial. For example, a red distractor signaled a high monetary payout for a correct and fast response for that trial, whereas a blue distractor signaled a low reward payout. Incorrect responses entailed a loss of an otherwise earned reward. Consistent with the classic finding of the additional singleton paradigm, participants were slower to respond to the target in the presence of the colored distractor that was never task-relevant. This behavioral pattern is commonly interpreted as involuntary capture of attention by the task-irrelevant distractor (Theeuwes, 2010). Crucially, however, Le Pelley et al. found that attentional capture by the distractor was greater when the distractor signaled the availability of a high relative to a low reward. Note that this erroneous capture of attention due to the distractor was maladaptive as slow responses to the target resulted in a loss of the reward.

The finding of attentional capture by reward-associated distractors, which were always task-detrimental, suggests that attentional selection is biased toward *any* signal of reward. In a follow-up experiment by Le Pelley et al. (2015), participants engaged in an oculomotor version of the same paradigm, in which they were required to make a quick saccade to the target (shape singleton). If participants made an erroneous saccade to the reward-signaling distractor (color singleton), reward was omitted. Again, capture for colored distractors, measured in terms of the number of reward omissions, was larger when they signaled high relative to low reward, supporting the idea that selection biases toward reward signals are not solely due to instrumental response-shaping. Instead, the authors argued that the reward signal alone must have influenced the degree to which a stimulus elicited attentional or oculomotor capture (Le Pelley et al., 2016).

However, in Le Pelley et al.’s (2015) paradigm, the colored distractor was always physically salient. It is well-documented that salient stimuli “pop out,” capturing attention both covertly and overtly in a reflexive manner (Theeuwes, 1992, 1994; Theeuwes, Kramer, Hahn, Irwin, & Zelinsky, 1999; Yantis & Egeth, 1999). In Le Pelley et al. (2015), the stimulus that signaled reward popped out because it was always the only colored stimulus in the display. It is therefore likely that initial capture was driven by the physical salience of the stimulus and not by the reward it signaled. Consequently, this raises the possibility that if the stimulus that signals reward would not have been physically salient, no attentional capture would have been observed.

To test this idea, Failing et al. (2015) conducted an experiment in which the reward-signaling distractors were no longer physically salient. Their search display was identical to Le Pelley et al. (2015) with the exception that now all stimuli were colored. Participants were informed prior to the start of the experiment that the presence of a particularly colored nontarget shape (e.g., red circle) would signal the availability of a relatively high reward while another particularly colored nontarget shape (e.g., blue circle) would signal the availability of a low reward for a correct and quick response. Participants were instructed to ignore this reward-signaling distractor and informed that if they moved their eyes to it, reward would be omitted even if they subsequently managed to make a saccade to the target on time. Even though the reward-signaling stimuli were task-irrelevant and physically nonsalient, there was still significantly more oculomotor capture by a distractor that signaled high relative to low reward. In other words, competition for selection between the reward-signaling distractor and the target occurred even though reward was immediately omitted when participants’ gaze fell on the reward-signaling stimulus. Importantly, this maladaptive behavior was particularly evident in for short-latency (i.e., rapidly initiated) first saccades, suggesting that reward-signaling stimuli involuntarily capture the eyes at an early stage in the visual selection process, independently of whether they are or were ever task-relevant or physically salient.

Previous studies provide evidence for a strong influence of reward on visual selection. Studies have shown that relatively high reward in the form of an incentive cue promotes cognitive control, increasing overall performance in a variety of visual tasks. Conversely, studies have shown that the bias of cognitive control in favor of stimuli associated with high reward comes at a cost, hampering task performance when such stimuli are not task-relevant. This interference in performance by stimuli signaling reward cannot be sufficiently accounted for by other known top-down or bottom-up processes (Awh et al., 2012). Although the two different ways by which reward affects attentional selection (i.e., beneficial motivational control vs. adverse distractor interference) have been topics of intensive research, they have been studied largely in isolation. As a consequence, there is little understanding over the interplay between and the circumstances under which these different ways by which reward affects attentional selection interact with each other.

The present series of experiments was designed to further investigate under what conditions stimuli that merely signal the availability of reward either improve performance or interfere with selection. Participants covertly searched for a target defined by either an outline color or shape. Across different experiments, non-target stimuli signaling the magnitude of potential reward (high or low) were presented either before or during the presentation of the search display.

In Experiments 1, 2, and 3 we demonstrate that a reward-signaling stimulus can, in some conditions, have a beneficial effect on task performance, while such a stimulus involuntarily captures attention and thus interferes with a given task in other conditions. Experiment 4 establishes that attentional capture of a task-irrelevant and nonsalient reward signal is characterized by a learning pattern. Experiment 5 further investigates the contribution of knowledge about the stimulus-reward association in this form of attentional capture. Finally, Experiment 6 provides evidence for a crucial role of awareness of the stimulus-reward association in learning the association and thereby prioritizing attentional selection of reward-signaling but otherwise task-irrelevant and non-salient stimuli.

## Experiment 1: Facilitation and interference in attentional selection due reward-signaling stimuli

Experiment 1 was designed to establish whether attentional selection is impacted by the presence of a nonsalient and task-irrelevant stimulus that merely signals the availability of a relatively high compared with low reward. In particular, we investigated the conditions in which such stimuli would be beneficial or detrimental to attentional selection. Experiment 1 consisted of two sessions in which participants were required to search covertly for a specific colored circle presented among other differently colored nontarget circles. Correct responses that were fast enough were rewarded. In the first session, participants were informed that a tone presented before the onset of the search display signaled the availability of either a high or a low reward for that particular trial.

**Fig. 1:**
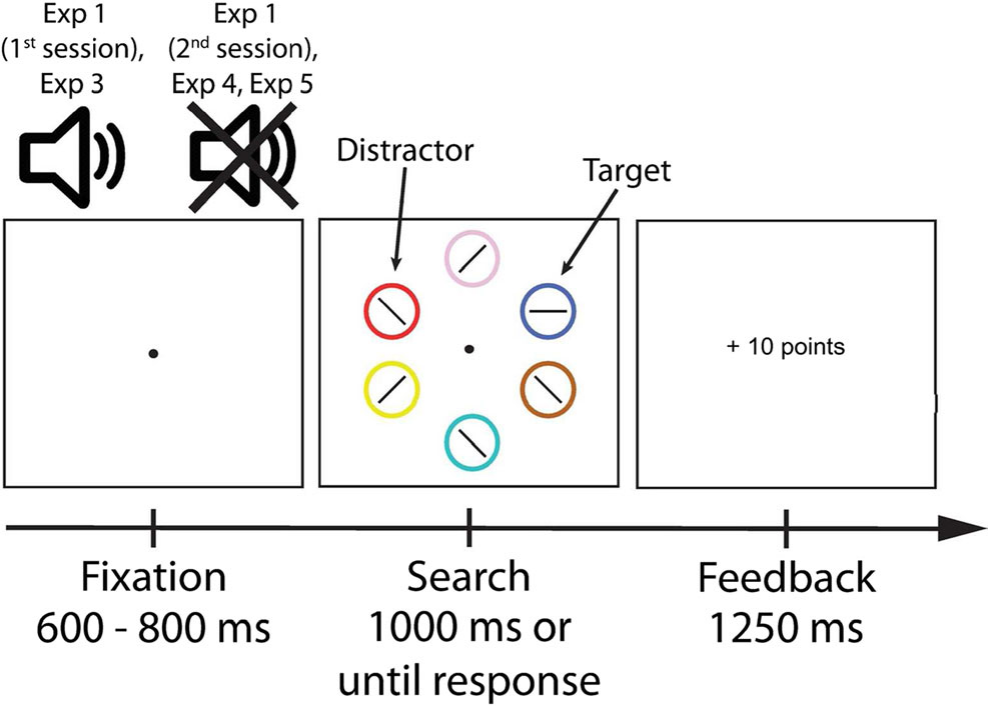
Trial sequence of Experiments 1, 3, 4, and 5. Participants were instructed to search for a particularly colored (e.g., blue) target circle and report the orientation of a line segment within the target. During the first session of Experiment 1, participants were informed that one of two different tones presented before the search display signaled whether high or low reward could be earned for a correct and quick response in that particular trial. These tones were omitted during the second session. During both sessions, the appearance of a particularly colored distractor circle (e.g., red) signaled that a high reward could be earned, whereas the appearance of a differently colored circle (e.g., green) signaled a low reward. Crucially, only during the second session were participants informed about the color-reward relationship. For Experiment 3, the sequence was similar to the first session of Experiment 1 with the exception that participants were now told about both stimuli (tone and colored circle) signaling reward. For Experiment 4, the sequence was also similar to the second session of Experiment 1, except that the relationship between the particularly colored distractor circles and the reward magnitude (high or low) was swapped every block. For Experiment 5, the sequence was also similar to the second session of Experiment 1, except that participants received no information about the color-reward relationship. Note that the ISI of 250 ms between the search display and feedback is not shown

In the second session, no tones were presented. Instead, reward magnitude was exclusively signaled by the presence of a particularly colored non-target circle in the search display (e.g., a red circle would signal a high reward; a green circle would indicate a low reward). As in the first session, participants were informed about the reward-association and that reward was only given if the response was fast enough. This was done to emphasize that attentional selection of the reward-signaling stimulus in the second session would only slow down responding and thus reduce the likelihood of reward payout. Crucially, because the display consisted of multiple colored circles, the circle signaling the reward availability was nonsalient (Fig. 1). Note that the colored nontarget circle signaling reward was not only present in the second but also the first session. However, its association with reward was only made explicit at the start of the second session.

We expected that prioritization of a non-target stimulus signaling relatively high reward presented before the onset of the search display (first session) would motivate participants to perform particularly well resulting in faster search times for high relative to low reward trials. For the second session, however, we expected the complete opposite result: if participants would be captured by the stimulus signaling the reward availability they should be slower in finding the target when there was a stimulus signaling a high relative to a low reward.

### Material and methods

#### Participants

Eighteen students from the Vrije Universiteit Amsterdam (14 females, mean age ± 22) with reported normal or corrected-to-normal vision participated in Experiment 1. All participants were naïve as to the purpose of the experiment. This and all following experiments were approved by the local ethics committee of the Vrije Universiteit Amsterdam. Participants received monetary compensation of between €8 and €12 (*M* = €8.40 ± *SD* = 0.50) based on performance.

#### Apparatus and stimuli

Participants were seated in a sound-attenuated dimly lit room with their heads on a chin rest at a distance of 70 cm from the screen. The experiment was separated into two sessions that took place on the same day. Stimuli used in both sessions were created in OpenSesame v2.8 (Mathôt, Schreij, & Theeuwes, 2012) and presented on a Samsung SyncMaster 2233RZ monitor (1,680 × 1,050 resolution, 120-Hz refresh rate). The search display consisted of six outline circles (3° visual degrees diameter) each of which was uniquely colored in one of seven colors (blue, CIE: x = 0.148, y = 0.096, 9.2 cd/m^2^; red, CIE: x = 0.620, y = 0.364, 28.0 cd/m^2^; green, CIE: x = 0.263, y = 0.651, 63.2 cd/m^2^; yellow, CIE: x = 0.425, y = 0.522, 93.2 cd/m^2^; pink, CIE: x = 0.350, y = 0.291, 48.5 cd/m^2^; brown, CIE: x = 0.444, y = 0.523, 16.1 cd/m^2^; cyan, CIE: x = 0.193, y = 0.388, 75 cd/m^2^) and presented on a black background. Each search display contained exactly one target circle, which for each participant had a particular color (e.g., blue) drawn from a pool of three different colors (red, green, or blue). Each display contained exactly one non-target circle that was colored in one of the two remaining colors from the pool (i.e., green or red). The circles were presented at equal distances on an imaginary circle (6.5° radius) around a white fixation dot. Within each circle was a line segment (1.8°) that could have one of four different orientations depending on whether it was inside the target circle (0° or 90° angular degrees tilted from vertical) or not (45° or 135°). In the first session, the onset of the search display was preceded by the presentation of a pure tone with either a high or low frequency (1,000 Hz or 500 Hz).

#### Procedure and design

##### First session

Each trial of the first session consisted of a fixation display, search display, blank ISI and feedback display (Fig. 1). A trial started with a randomly determined fixation period of 400-600 ms followed by an auditory cue presented for 200 ms while the fixation screen remained visible. Following the auditory cue, the search display—consisting of six uniquely colored circles—was displayed for 1,000 ms or until response. Participants were instructed to indicate the orientation of a line segment inside the target circle by pressing the appropriate key (‘X’ for 0° tilt; ‘M’ for 90° tilt). During both sessions, the target circle was defined by a specific color. Following a response or timeout and an ISI of 250 ms, the feedback display was shown for 1,250 ms. For a correct and quick (see below) response, this feedback showed a “+” sign and the number of points participants had earned for that trial. Correct and quick responses were always rewarded 10 points in high reward trials and 1 point in low reward trials. For responses that were too slow, participants earned no reward, and for incorrect responses, participants lost the amount of points they would have earned if their response had been correct (denoted by a “−” sign and the number of points lost). Participants were informed that the points corresponded to real money and that they could earn up to €12 paid out at the end of the experiment. No information was provided about how many points corresponded to how much money.

Two design features were important in the first session: First, the occurrence of each tone (high or low frequency) was accompanied by the presence of a specifically colored nontarget circle, the reward-signaling distractor, in the search display. The colors of that nontarget circle were selected from a pool of three different colors (red, green, blue) while the remaining color of that pool defined the target circle. Both, the tone (high or low) and the specifically colored nontarget circle (e.g. red or green), signaled that either high or low reward could be earned for a correct and quick response. For example, for one participant, the target circle could be colored in blue, whereas a high tone and the presence of a red distractor circle signaled a high reward, and a low tone and the presence of a green distractor circle signaled a low reward (Fig. 1). The reward magnitude signaled by the tone and the colored nontarget circle was always the same. Importantly, however, in the first session participants were only informed about the relationship between the tone and the reward (i.e., which tone signaled which reward) but were told to ignore the tone and to focus on searching for the target. Note that both tone-reward and color-reward relationships were individually counterbalanced across all participants. Second, to keep participants motivated to respond quickly, a variable response time (RT) limit was implemented. For each participant, this variable RT limit was based on the 75^th^ percentile of all individual RTs in the preceding block. If participants responded slower than the variable RT limit, they were still able to indicate their response until the trial timed out but would no longer receive any reward for a correct response.

Each participant performed one practice block and five experimental blocks of 60 trials each, yielding a total of 360 trials in the first session. Half of the trials in each block featured a high-frequency tone and one specifically colored distractor, and the other half featured a low-frequency tone and the remaining specifically colored distractor.

##### Second session

The trial sequence of the second session differed in only two aspects from the first session. First, no auditory cue was presented during the fixation period (Fig. 1). Second, participants were informed about the relationship between the presence of a specifically colored circle and the reward that could be earned for a correct and quick response (e.g., that the presence of a red circle in the display signaled that high reward was available, and a green circle signaled low reward). The colors that signaled either a high or low reward for a correct and quick response were the same as during the first session. Participants were instructed at the start of the session to ignore these colored circles, and it was emphasized that attending them would most likely lead to no reward payout or a loss of already earned reward.

Each participant performed one practice block and five experimental blocks of 60 trials each, yielding a total of 360 trials in the second session. Half of the trials in each block featured one specifically colored distractor and the other half featured the remaining specifically colored distractor.

#### Data analysis

For the RT analyses only correct responses were used. Responses faster than 200 ms were discarded. No other data were excluded from the RT analyses. Note that all *p* values are Greenhouse-Geisser corrected even though unadjusted degrees of freedom are reported. These data analysis criteria were used here as well as in the analyses for all the other experiments.

### Results

**Fig. 2:**
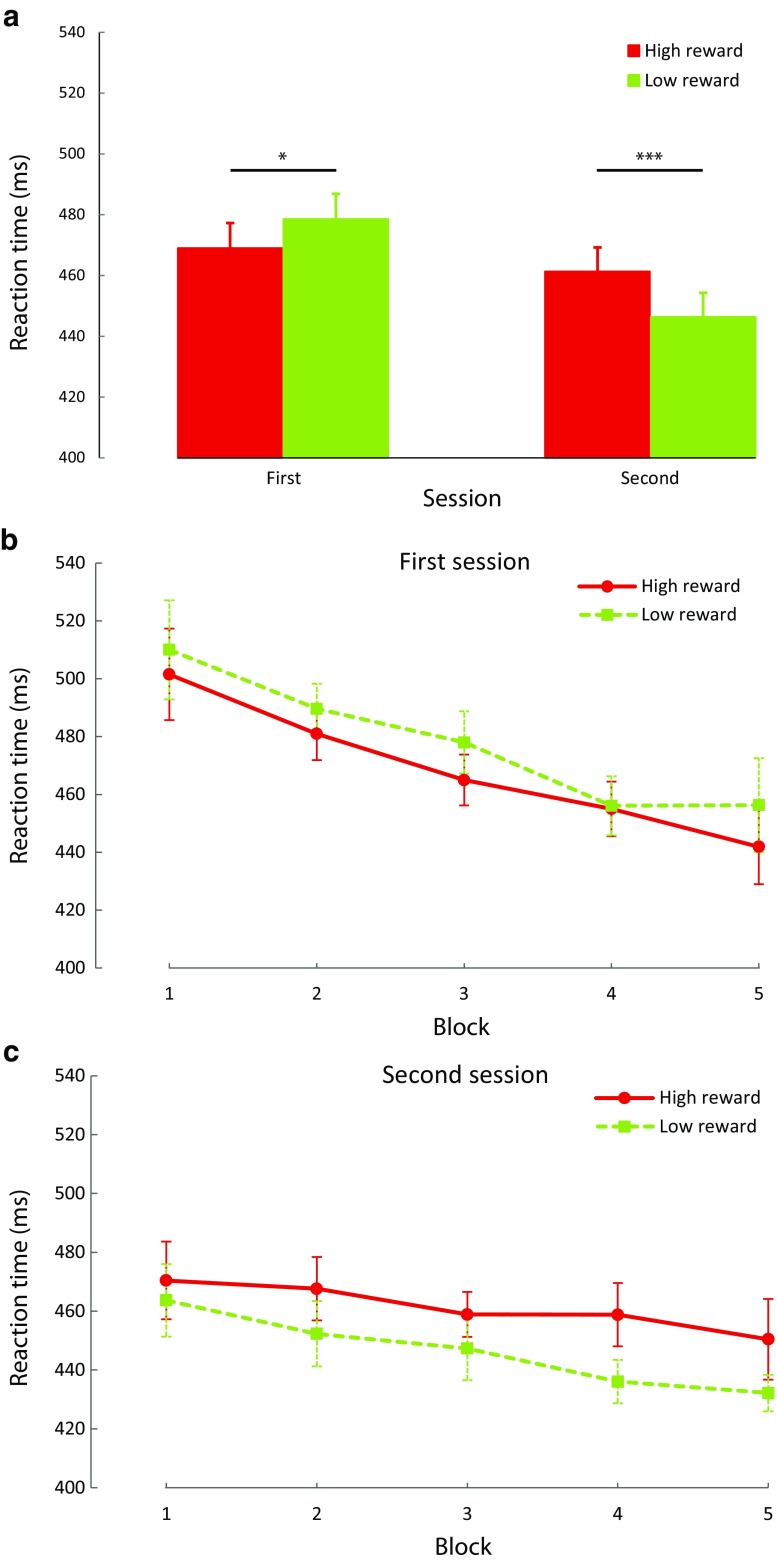
Results of Experiment 1. (**a**) Mean reaction time by reward condition over sessions. (**b**) Mean reaction time by reward condition over all blocks of the first session. (**c**) Mean reaction time by reward condition over all blocks of the second session. These graphs illustrate that the reward effect flipped from the first to the second session. In the first session, mean reaction time was significantly quicker for high- compared with low-reward trials. In the second session, the opposite pattern was observed with a significantly slower mean reaction time for high- compared with low-reward trials. Error bars represent within-subject 95% confidence intervals (Cousineau, 2005; Morey, 2008) and ***p < 0.001, **p < 0.01, *p < 0.05, ^†^ *p* < 0.10, n.s. *p* > 0.10 here and in all other figures

To examine whether attentional selection would be differentially impacted by the presentation of a reward-signaling stimulus before or during the search display, we ran a within-subjects analysis of variance (ANOVA) on individual mean RT, with factors of session (first vs. second) and reward (high vs. low). Our primary hypothesis was that the impact of the reward signal would flip from facilitation to interference across session. In line with this hypothesis, there was a highly reliable interaction, *F*(1,17) = 19.424, *p* < 0.001, η^2^ = 0.533 (Fig. 2a). During the first session, participants responded faster when the stimulus signaled a high reward (*M* = 469 ms ± *SD* = 50) relative to low reward (478 ms ± 54), *t*(17) = 2.440, *p* = 0.026, 95% CI [1.30, 17.92]. In contrast, during the second session, participants were slower when the stimulus signaled a high reward (461 ms ± 54) compared with a low reward (446 ms ± 51), *t*(17) = 3.888, *p* = 0.001, 95% CI [6.76, 22.80]. There was no main effect of reward, *F*(1,17) = 0.959, *p* = 0.341, but session was marginally significant, *F*(1,17) = 4.136, *p* = 0.058, η^2^ = 0.196.

To assess whether the reward effect changed over the course of each session we submitted RT data to an ANOVA across factors of session (first vs. second), reward (high vs. low) and block (1-5). The results showed a marginally significant main effect of session, *F*(1,17) = 4.009, *p* = 0.062, η^2^ = 0.191, a main effect of block, *F*(4,68) = 22.390, *p* < 0.001, η^2^ = 0.566, but not of reward, *F*(1,17) = 1.204, *p* = 0.288. Neither the interaction of reward and block, *F*(4,68) = 2.124, *p* = 0.108, nor the interaction of session, reward and block reached significance, *F*(4,68) = 0.851, *p* = 0.478. However, the interaction of session and block, *F*(4,68) = 5.086, *p* = 0.005, η^2^ = 0.230, and the interaction of session and reward reached significance, *F*(1,17) = 18.379, *p* < 0.001, η^2^ = 0.519. This suggests that while there was a general decrease in mean RT over blocks which also differed for both sessions, the difference in the reward effect did not differ significantly over the course of each session (Fig. 2b and c).

To rule out a potential speed-accuracy tradeoff explanation of reward effects, we performed a similar analysis on error rates, finding only a significant main effect of session, *F*(1,17) = 8.539, *p* = 0.010, η^2^ = 0.334, and block, *F*(4,68) = 6.416, *p* < 0.001, η^2^ = 0.274. None of the other effects were significant (all *p* > 0.05). This demonstrates that while participants made fewer errors over the course of the blocks and generally more errors in the second session (high vs. low: 10.1% vs. 10.1%) compared with the first session (high vs. low: 7.6% vs. 7.9%), the influence of reward on mean RT cannot be explained in terms of a speed accuracy trade-off in either of the two sessions.

We hypothesized that any interference by reward-signaling stimuli in the second session would occur even though the interference was detrimental to actual reward payout. To assess whether this was indeed the case, we compared the number of trials that were faster than the variable RT limit. A paired samples *t* test confirmed our hypothesis showing that there were significantly fewer trials faster than the RT limit in the high compared with the low reward condition during the second session, *t*(17) = 2.039, *p* = 0.001, 95% CI [4.87, 16.24]. In other words, participants indeed missed out more often on high than low reward payout during the second session of Experiment 1.

### Discussion

Results from the first session of Experiment 1 show that a stimulus signaling a high potential reward facilitates performance when presented before the search display. This effect is consistent with many previous studies showing that participants are motivated to perform better on a given task when a high relative to a low reward can be earned (Bucker & Theeuwes, 2014; Engelmann & Pessoa, 2007; Pessoa & Engelmann, 2010; Small et al., 2005). Importantly, because the reward signal was fully orthogonal to the correct response, the observed performance benefits could not be the result of improved response preparation. Instead, they were likely due to a stronger top-down set to perform better on a trial for which a high relative to low reward can be earned (Sawaki et al., 2015). The finding that a reward signal for relatively high reward improves performance when it is not competing with the target (e.g., by not being presented in the same display as the target) is consistent with the idea that reward used as an incentive fosters cognitive control (Botvinick & Braver, 2015).

Results from the second session of Experiment 1 demonstrate that—with the same task demands as the first session—a stimulus that signals the availability of reward not before but *within* the search display has a completely opposite effect on selection. Indeed, performance declined (i.e., search time increased) when the search display contained a stimulus that signaled a high relative to a low reward. This effect can be explained in terms of attentional capture: the stimulus that signaled high reward captured attention more often than a stimulus signaling a low reward.

It is crucial to note that unlike in previous studies (Le Pelley et al., 2015), the reward-signaling distractor stimulus in the current study did not stand out from the other (nontarget) stimuli, as the search display consisted of six circles each having a unique color (Fig. 1). It is well-known that physically salient stimuli “pop-out,” and this pop-out effect has been shown to cause interference in visual search independent of the current task set or task-relevance, a finding typically explained in terms of involuntary capture of attention (Theeuwes, 1992, 1994; Yantis and Egeth, 1999). This raises the possibility that—for stimuli that have never been task-relevant—reward learning is unable to drive attentional capture in its own right but merely modulates the degree of capture that is otherwise driven by physical salience. The findings from the second session of our Experiment 1, however, demonstrate that physical salience is not necessary for a task-irrelevant but reward-signaling stimulus to capture attention.

Importantly, the reward-signaling distractor stimulus was never relevant for the task and participants were not instructed to select it during a training session, which often is seen in these type of experiments (Anderson, 2013; Chelazzi et al., 2013). In fact, selecting the reward-signaling distractor stimulus in the second session was detrimental to task performance, because it increased the likelihood of omission or loss of otherwise earned reward. The current effects are quite different from studies that have shown attentional capture only after an exhaustive training session in which the selection of that reward-signaling stimulus was either necessary for successful task performance, or accomplished through other, involuntary, forms of attentional selection (e.g., attentional capture by physical salience).

The second session of this experiment has many similarities to Failing et al. (2015). Similar results were observed as the task-irrelevant and physical nonsalient but reward-signaling stimuli elicited oculomotor capture. Nonetheless, it should be noted that a replication of this effect in the context of covert search is not superfluous as there may be a dissociation between attentional and oculomotor capture in some cases (Belopolsky & Theeuwes, 2009; Theeuwes, De Vries, & Godijn, 2003).

## Experiment 2: Non-salient and task-irrelevant stimuli signaling reward capture attention independently of the task set

It is possible that the reward-signaling stimulus captured attention during the second session of Experiment 1, because it was defined within the same visual feature domain as the target (i.e., color). In other words, because participants were looking for a particular color (i.e., the color defining the target), a general attentional set for color may have caused attention to be allocated selectively to the color that signaled the availability of reward (Bacon & Egeth, 1994). To rule out this possibility, we conducted a follow-up experiment to the second session of Experiment 1 in which the target was defined by a stimulus feature that was different than the one that signaled the reward. In Experiment 2, the target was defined as a shape singleton while a particularly colored, yet nonsalient, nontarget in the search display signaled the amount of reward available during that trial.

### Materials and methods

#### Participants

Eighteen students from the Vrije Universiteit Amsterdam (11 females, mean age ± 22 years) with reported normal or corrected-to-normal vision participated in Experiment 2. All participants were naïve as to the purpose of the experiment and had not participated in Experiment 1. Participants received monetary compensation of between €6 and €10 (€6.70 ± 0.90) based on performance.

#### Apparatus and stimuli

**Fig. 3:**
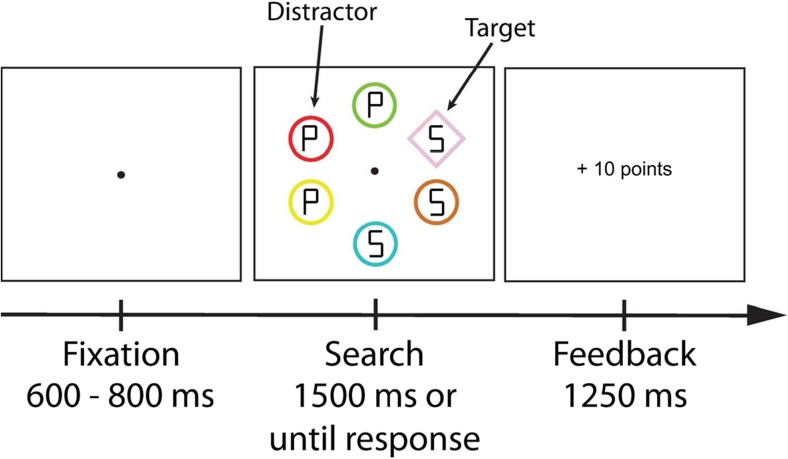
Illustration of the trial sequence of Experiment 2. Participants were instructed to search for the shape singleton (target; e.g., diamond among circles) and report the identity of a letter within the target. The appearance of a particularly colored shape (distractor; e.g., red circle) signaled that a high reward could be earned while the appearance of a differently colored shape (e.g., blue circle) signaled a low reward. Participants were informed about the color-reward relationship but were instructed to ignore it and to focus on searching for the target shape. Note that the ISI of 250 ms between the search display and feedback is not shown

The experimental setup was similar to that of the second session of Experiment 1. The experimental task, however, was based on the additional singleton paradigm (Theeuwes, 1991, 1992). The search display consisted of six outline shapes: either one diamond (3.2° by 3.2° visual degrees) and five circles (3° diameter) or one circle and five diamonds, each of which was uniquely colored (red, blue, green, yellow, pink, brown, or cyan). Each search display contained exactly one nontarget shape of a specific color (red or blue). Inside of each outline shape was one of two letters (“S” or “P”; 1.65° by 0.82°, made up of equally-sized line segments; Fig. 3).

#### Procedure and design

The procedure and design were similar to the second session of Experiment 1 with several exceptions. The target in the search display was defined by the shape singleton (diamond or circle). Participants were instructed to report the letter inside that shape singleton (using the X key for the letter “S”; and the M key for the letter “P”). As in Experiment 1, the presence of a particularly colored nontarget shape (blue or red; counterbalanced over participants) among the differently colored non-target shapes signaled either high reward or low reward (Fig. 3). The shape singleton was never rendered in either of the reward-signaling colors (red or blue). Because these changes made the task more demanding, we anticipated generally higher reaction times and therefore increased the presentation time of the search display to 1,500 ms.

As in Experiment 1, participants in Experiment 2 were explicitly informed about the relationship of each reward-signaling distractor stimulus and its corresponding reward payout. They also were instructed to ignore these reward-signaling stimuli, because attending to them would most likely lead to the omission of reward or a loss of reward already obtained. Participants were informed prior to the experiment that they could earn up to €10 depending on the number of points they acquired throughout the experiment. As in Experiment 1, no information was given about how many points corresponded to how much money.

Each participant performed one practice block and six experimental blocks of 80 trials each, yielding a total of 560 trials. Half of the trials in each block featured a red distractor and the other half a blue distractor. Likewise, half of the trials in each block featured a diamond and the other half a circle as the target shape.

### Results

To investigate whether the reward-signaling distractor colors affected search times to the shape singleton target, we first compared the mean RT of both reward conditions. A paired-samples *t* test showed a significant difference for high reward compared to low reward trials, *t*(17) = 2.409, *p* = 0.028, 95% CI [1.43, 21.66]. Participants responded on average slower in high reward (756 ms ± 85) than in low reward trials (745 ms ± 83). A subsequent analysis showed that participants also missed out more often on reward payout in the high than in the low reward condition, (*t*(17) = 2.260, *p* = 0.037, 95% CI [.37, 10.85].

**Fig. 4:**
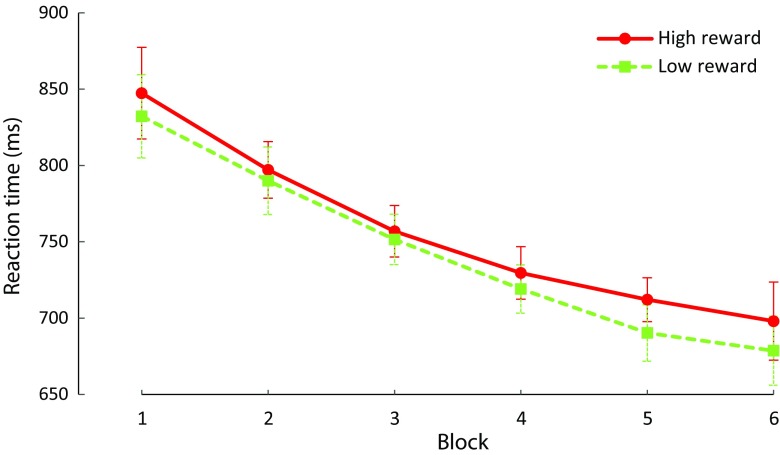
Results of Experiment 2. Mean reaction time by reward condition over all blocks. Reaction times were significantly slower on high- compared with low-reward trials

To assess whether the reward effect changed over time, we ran an ANOVA on mean RT using reward (high vs. low) and block (1-6) as factors. There was a main effect of reward, *F*(1,17) = 8.541, *p* = 0.009, η^2^ = 0.334, and block, *F*(5,85) = 42.019, *p* < 0.001, η^2^ = 0.712, but no interaction, *F*(5,85) = 0.826, *p* = 0.510. RT exhibited a linear trend over the course of the experiment, *F*(1,17) = 70.769, *p* < 0.001, η^2^ = 0.806, indicating that mean RT generally decreased over the course of the experiment for both reward conditions. Again, this suggests that the reward effect remained stable over the course of Experiment 2 (Fig. 4). There were no reliable differences in error rates (all *p* > 0.05; high vs. low: 21.5% vs. 21.3%).

### Discussion

Experiment 2 replicated the main findings of the second session of Experiment 1. When the task-irrelevant distractor signaling high reward was present in the search display, it caused more interference even though it did not stand out from the display (i.e., it was nonsalient). The interference occurred even though the target was defined by a different visual feature than the one signaling reward. This suggests that when searching for one particular feature (here shape), a different feature that has never been part of the task set but signals the available reward (here color) still captures attention. The optimal strategy in Experiment 2 was to simply ignore any color and reward information in order to perform well on the task and consequently increase reward payout. Yet, even though participants were informed about this strategy and instructed to use it, the results indicate that they could not do so. Clearly, the reward-signaling stimulus captured attention even though a better strategy was available.

In conjunction with the first session of Experiment 1, these findings extend previous findings (Le Pelley et al., 2015; Pearson et al., 2015) by demonstrating conclusive evidence for attentional capture by reward-signaling stimuli that cannot be explained in terms of either top-down task-relevance, task set or bottom-up salience.

## Experiment 3: Attentional capture by reward-signaling stimuli occurs only when the reward signal is informative

The second session of Experiment 1 demonstrated attentional capture by stimuli in the search display that merely signaled reward. In contrast, during the first session of Experiment 1, participants were more efficient in selecting the target stimulus due to the high-reward signaling stimulus presented prior to the search display. Importantly, facilitation occurred even though the reward-signaling distractor that interfered with search during the second session was also present in the search display during the first session. In other words, another potentially important difference between the first and second session of Experiment 1 was that participants were either informed about the reward-signaling property of a stimulus in the search display (second session) or not (first session). The influence on search performance exerted by the reward-signaling stimulus in the search display may therefore also be explained in terms of the information participants received about which stimuli reliably predict the reward magnitude on a particular trial. Alternatively, it may be that if the information conveyed by the reward-signaling stimulus in the search display is redundant, because it is already conveyed by the reward-signaling stimulus presented prior to the onset of the search display, a reward-signaling stimulus in the search display does not capture attention because participants are simply able to ignore it.

Experiment 3 investigated whether a stimulus signaling the availability of reward still competes for attentional selection when the information that this stimulus conveys is also available through other sources provided earlier in time. To that end, we combined the two sessions of Experiment 1. Participants were informed that a tone presented prior to the onset of the search display and one of the colored circles in the search display would signal the reward that could be earned on that trial. If a reward-signaling stimulus only gains priority when it is the *only* stimulus that signals availability of reward, the reward-signaling stimulus in the search display should no longer compete for attention as its reward signal is rendered redundant. Consequently, we would expect facilitation of target search for high relative to low reward trials due to the reward-signaling stimulus presented prior to the search display. This would correspond to a replication of the incentive motivation effect observed in the first session of Experiment 1. Conversely, if reward-signaling stimuli always gain priority due to actively informing participants about the relationship between the stimuli and the reward they signal, both effects should counteract each other leading to a diminished or even the absence of a significant reward effect.

### Materials and methods

#### Participants

Another 18 students from the Vrije Universiteit Amsterdam (12 females, mean age ± 22) with normal or corrected-to-normal vision participated in Experiment 3. All participants were naïve as to the purpose of the experiment and had not participated in any of the previous experiments. Participants received monetary compensation of between €5 and €10 (€7.80 ± 1.00) based on their performance.

#### Apparatus and stimuli

The experimental setup was similar to the first session of Experiment 1.

#### Procedure and design

The procedure and design were similar to the first session of Experiment 1 with one exception. Participants were informed that both the tone and the presence of a particularly colored circle in the display would signal the reward payout for quick and accurate target discrimination (Fig. 1). They also were instructed to ignore both the tone and the colored distractor that signaled reward and to focus entirely on the search for the target circle. Before the experiment, participants were informed that they could earn up to €10 depending on the number of points they acquired throughout the experiment.

Each participant performed 60 trials of practice followed by 6 experimental blocks with the same amount of trials, yielding a total of 420 trials. Half of the trials in each block featured a high-frequency tone and one distractor rendered in the high reward color, and the other half featured a low-frequency tone and the one distractor rendered in the low reward color.

### Results

To address the question whether a reward-signaling stimulus would capture attention if its reward signal is redundant, we ran a paired-samples *t* test comparison on mean RT for both reward levels (high vs. low). A reliable difference indicated that participants responded faster on high-reward (474 ms ± 50) than low-reward trials (488 ms ± 51), *t*(17) = 5.191, *p* < 0.001, 95% CI [8.22, 19.48].

**Fig. 5:**
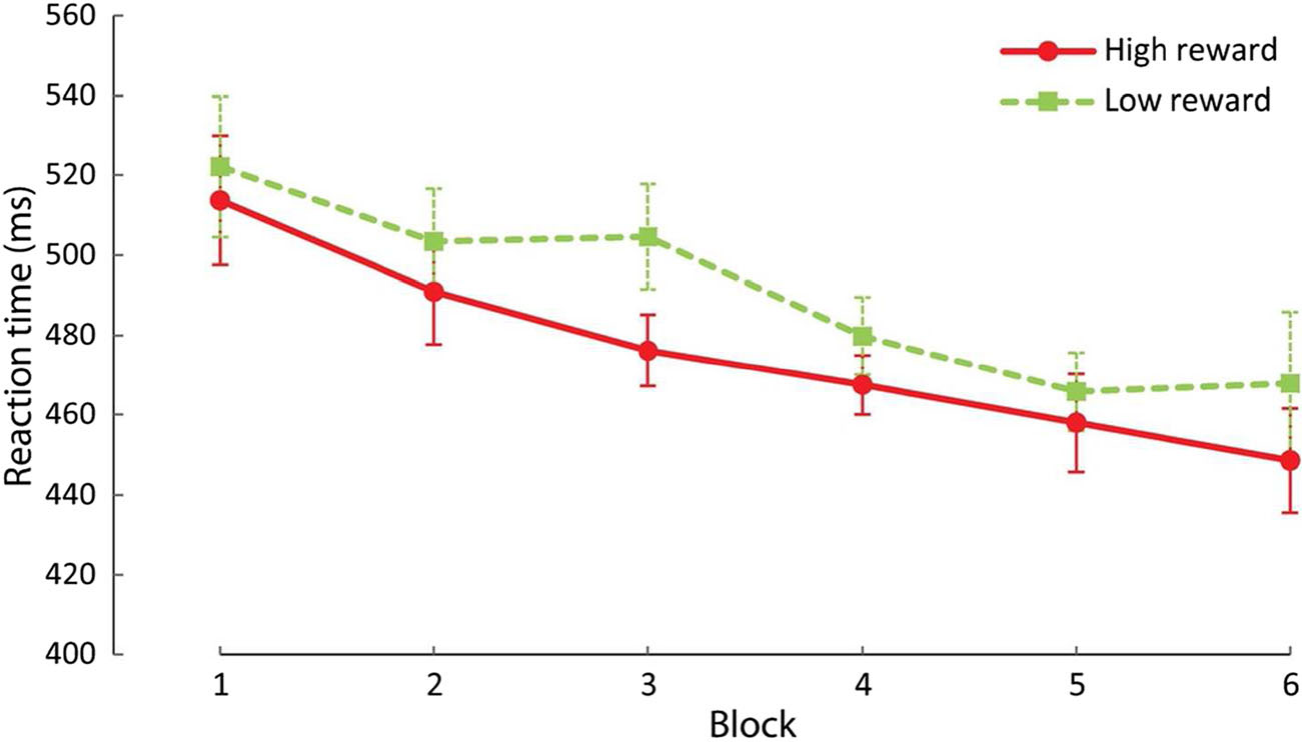
Results of Experiment 3. Mean reaction time by reward condition over all blocks. Reaction times were significantly faster on high- compared with low-reward trials

An ANOVA on mean RT using reward (high vs. low) and block (1-6) as factors assessing whether the reward effect changed over time showed a significant main effect of reward, *F*(1,17) = 23.852, *p* < 0.001, η^2^ = 0.584, and block, *F*(5,85) = 22.759, *p* < 0.001, η^2^ = 0.572, but no interaction, *F*(5,85) = 1.119, *p* = 0.352. Mean RT exhibited a linear trend over the course of the experiment, *F*(1,17) = 30.470, *p* < 0.001, η^2^ = 0.642, which indicates that participants responded faster over time irrespective of the reward condition. Again, the results also suggest that the reward effect remained relatively stable over the course of Experiment 3 (Fig. 5).

A similar analysis on error rates showed a significant main effect of block, *F*(5,85) = 9.482, *p* < 0.001, η^2^ = 0.358, but no main effect of reward or interaction (*p* > 0.05). Error rates remained generally low (high vs. low: 9.9% vs. 9.9%). This shows that the decrease in RT cannot be explained in terms of a speed-accuracy trade-off.

### Discussion

In Experiment 3, we observed a similar effect as in the first session of Experiment 1: search times were faster for high- than low-reward trials. This demonstrates that participants could ignore the stimulus in the search display indicating the amount of reward to be earned simply because they had already received the very same information through the tone presented before display onset. The reward-signaling stimulus presented before the onset of the search display affected search by acting as an incentive cue to motivate target search. This is consistent with the notion that reward in the form of an incentive cue can foster cognitive control (Botvinick & Braver, 2015).

Crucially, the finding of Experiment 3 suggests that informing participants about the presence of stimuli in the search display that signal the availability of reward does not automatically prioritize those stimuli. As a consequence, they are not more likely to be selected than any other nontarget in the search display, which explains why there was no consistent interference by the reward-signaling stimuli. These results are in line with the idea that a reward-signaling stimulus only captures attention if it is, at the time of onset, uniquely predictive of the reward, which can be earned on a given trial. This idea is consistent with findings from a recent study by Sali et al. (2014). They showed that only when stimuli uniquely predicted information about reward outcomes during a reward learning phase, they interfere as task-irrelevant distractors in target search during a subsequent reward-free test phase.

More generally, the procedure and the results of this experiment are reminiscent of what is known as “blocking” (Kamin, 1969). Blocking describes a mechanism by which pretraining with one part (say A) of a compound stimulus (say AB) prevents conditioning of a second part of a compound stimulus (say B). In the context of our experiment, one could speculate that informing participants about the predictive relationship of the tone (A) and the color (B) may be represent some form of pretraining. Because the tone is presented (and thus experienced) before the color, it may have blocked conditioning of the latter. Although such a mechanism also has been argued to hold true for attention (Mackintosh, 1975), it remains for further research to explore whether they can explain if and how reward-driven attentional selection occurs.

## Experiment 4: Attentional capture by reward-signaling stimuli occurs through learning

In the previous experiments, participants were informed that the color of one of the stimuli in the search display signaled the magnitude of reward that could be earned on that trial. The results showed that a task-irrelevant stimulus signaling a high reward caused more attentional capture than when it signaled a low reward but only if that stimulus was the only stimulus predicting reward outcome on that trial. Even though important, a question that remains unanswered is whether the explicit knowledge about the relationship between the stimulus and the reward is sufficient to observe attentional capture. In other words, is simply knowing the association the driving force or is the repeated association between the stimulus and the reward required to obtain this effect?

Both notions make explicit predictions. First, if capture is determined by knowledge about the stimulus-reward association, prioritization of a stimulus signaling availability of a high reward should occur instantaneously as soon as participants know about the relationship. Conversely, if the effect is determined by learning, prioritization should emerge, gradually adjusting to changes in the relationship between the stimulus and its signaled reward, which are experienced through iterative exposure.

In Experiment 4, we tested these predictions by changing the relationship between the stimuli and the magnitude of reward they signaled from block to block. During this experiment, the color-reward relationship between the stimuli signaling high and low reward was swapped every block. Participants were explicitly informed at the start of each block that the stimulus-reward association was changed for the upcoming block. If the interference by reward, as observed in the previous experiments, is only determined by knowledge about the association, we would expect that the reward effect quickly changes from one block to the next according to the information provided. As a consequence, attentional capture by the stimulus signaling availability of high reward should occur immediately, remain stable throughout the block and should not be affected by the reversed relationship of the previous block. Alternatively, if learning is important for attentional capture to occur, prioritization of the stimulus signaling high reward should more gradually emerge over the course of the block. A learning account also predicts that the effect of reward at the beginning of the block (i.e., immediately after the relationship swapped) should be most attenuated—if not reversed—due to the influence of the recent reward history from the previous block.

### Materials and methods

#### Participants

Another 18 students from the Vrije Universiteit Amsterdam (10 females, mean age ± 23) with reported normal or corrected-to normal vision participated in Experiment 4. All participants were naïve as to the purpose of the experiment and had not participated in any of the previous experiments. Participants received monetary compensation of between €8 and €14 (€10.55 ± 1.80) based on performance.

#### Apparatus and stimuli

The experimental setup was similar to the second session of Experiment 1.

#### Procedure and design

The experimental procedure and design of Experiment 4 were similar to the second session of Experiment 1 (Fig. 1). However, there were a few critical changes. In Experiment 4, the relationship between the distractor stimuli and the amount of the reward they signaled was swapped every block while the target stimulus remained the same throughout the entire experiment. For example, while the target remained a blue circle across all blocks, in the first block, the presence of a red circle signaled high potential reward and the presence of a green circle signaled low potential reward; in the subsequent block, red now signaled low reward and green now signaled high reward. Before the beginning of each block, participants were explicitly informed which colored circle would signal high reward and which would signal low reward in the upcoming block. It was emphasized that the target color would remain the same and that they should ignore the reward-signaling stimuli. Participants were furthermore informed that they could earn up to €14 depending on the amount of points they acquired throughout the experiment.

Each participant performed 60 trials of practice and 6 experimental blocks with 120 trials each, yielding a total of 780 trials. Half of the trials in each block featured one specifically colored distractor and the other half featured the remaining specifically colored distractor.

### Results

To assess whether we could replicate the interference effect of reward as observed in the previous experiments, we ran an ANOVA on mean RT using reward (high vs. low) and block (1-6) as factors. There was a significant main effect of reward, *F*(1,17) = 5.495, *p* = 0.031, η^2^ = 0.244, and block, *F*(5,85) = 28.326, *p* < 0.001, η^2^ = 0.625, but no interaction, *F*(5,85) = 0.751, *p* = 0.540. Participants were on average slower in high reward (503 ms ± 37) than in low reward trials (497 ms ± 34) and mean RT decreased linearly over blocks, *F*(1,17) = 39.757, *p* < 0.001, η^2^ = 0.700. For a similar analysis on error rate, only the main effect of block reached significance, *F*(5,85) = 3.346, *p* = 0.030, η^2^ = 0.164. There were no other reliable differences (all *p* > 0.05), indicating that RT differences cannot be explained in terms of a speed-accuracy trade-off. A subsequent analysis showed that participants also tended to miss out more often on reward payout in the high than in the low reward condition, although the difference in number of trials quicker than the RT limit did only reach marginal significance, *t*(17) = 1.908, *p* = 0.073, 95% CI [−0.89, 17.66].

To assess the time course of the reward effect throughout each block, we collapsed RT data over all blocks and separated it into six bins. Each bin contained 20 consecutive trials such that the first bin contained the first 20 trials of each block, the second bin contained the subsequent 20 trials of each block, and so on. Given that the order of trials was randomized within a block and only correct trials were used for the RT analyses, the total number of trials per reward condition per bin varied between 40-70 trials.

**Fig. 6:**
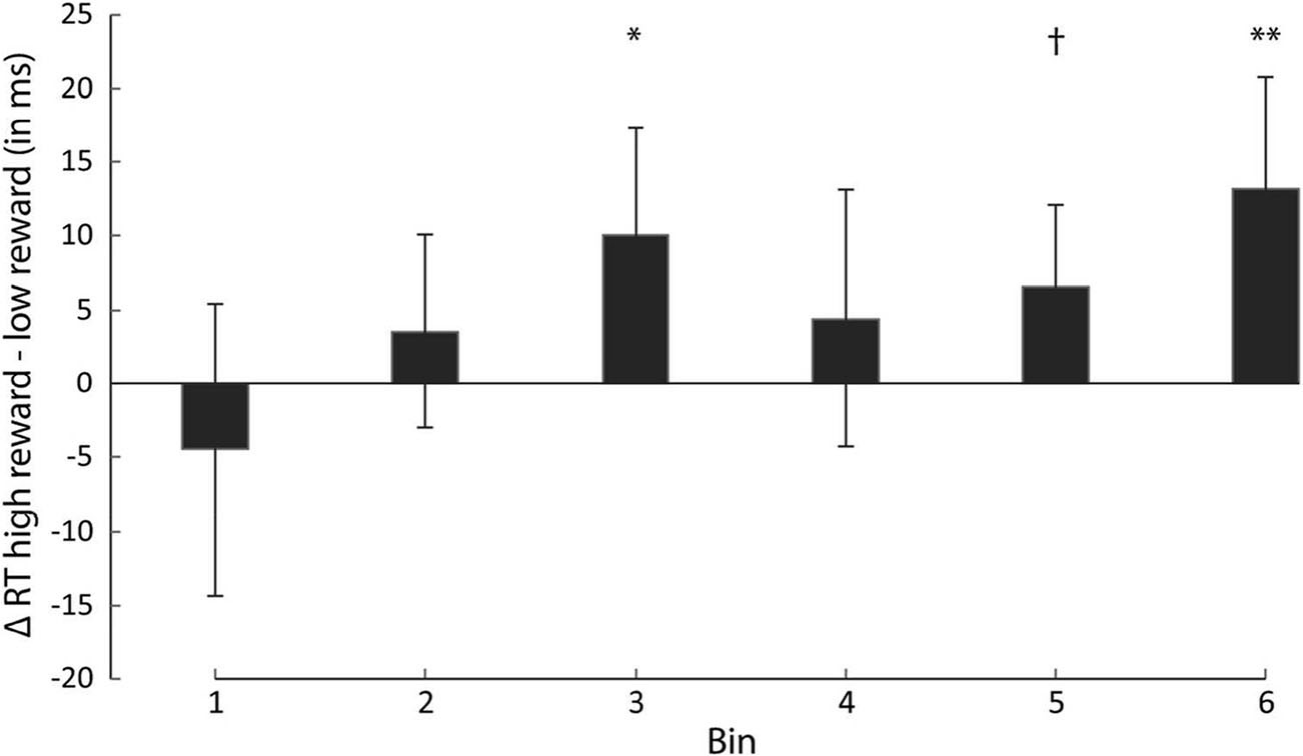
Results of Experiment 4. Difference scores of mean reaction time (RT) from high-reward—low-reward trials per bin. The difference in reaction time increased significantly over bins

An ANOVA on mean RT using reward (high vs. low) and bin (1-6) as factors showed a marginally significant main effect of reward, *F*(1,17) = 3.593, *p* = 0.075, η^2^ = 0.174, but no main effect of bin, *F*(5,85) = 1.823, *p* = 0.158. Crucially however, there was a significant interaction, *F*(5,85) = 2.914, *p* = 0.029, η^2^ = 0.146, which indicates that there was a significant change in the reward effect over bins. Figure 6 depicts the difference scores for both reward conditions (high reward - low reward) per bin. As illustrated by Fig. 6, mean RT for high-reward trials was numerically lower compared with low-reward trials in the first bin. The difference in mean RT for high-reward compared with low-reward trials reversed in the following bins with the difference tending to increase over subsequent bins. This linear trend was highly reliable, *F*(1,17) = 8.766, *p* = 0.009, η^2^ = 0.340, even though paired samples *t* test comparisons between the reward conditions did not reach significance in every bin (bin1: *t*(17) = 0.983, *p* = 0.339, 95% CI [−5.10, 14.00]; bin2: *t*(17) = 0.754, *p* = 0.461, 95% CI [6.36, 13.43]; bin3: *t*(17) = 2.579, *p* = 0.019, 95% CI [1.83, 18.25]; bin4: *t*(17) = 0.900, *p* = 0.381, 95% CI [−5.93, 14.75]; bin5: *t*(17) = 1.796, *p* = 0.090, 95% CI [−1.15, 14.31]; bin6: *t*(17) = 2.989, *p* = 0.008, 95% CI [3.87, 22.45]).

Analyzing error rates in the binned data revealed no statistically significant differences between high- and low-reward trials (all *p* > 0.05) and mean error rates in all bins were low (bin1: 10.1%; bin2: 9.0%; bin3: 9.3%; bin4: 10.7%; bin5: 9.0%; bin6: 10.8%), which indicates that these results cannot be explained in terms of a speed-accuracy trade-off.

### Discussion

Experiment 4 replicates the results from the previous experiments (Experiment 1 and 2) as participants responded slower on high- compared with low-reward trials overall, which demonstrates that the distractor in the search display signaling relatively high reward caused generally more interference in attentional search. Critically, however, this reward modulation was numerically reversed for the first bin within a block (i.e., right after the stimulus-reward association was swapped) and emerged gradually over the remaining bins with the strongest modulation observed in the last bin (i.e., before the stimulus-reward association would be swapped). This result is inconsistent with the view that the reward effect is solely driven by explicit knowledge regarding the stimulus-reward relationship. Instead the results indicate that attentional capture by a reward-signaling stimulus in the display is a consequence of learning through iterative exposure to the stimulus-reward association.

These findings are in line with the previous experiments reported in this study as well as others (Le Pelley et al., 2015; Pearson et al., 2015; Failing et al., 2015). Most of these studies, however, could not provide clear evidence for learning of the stimulus-reward association aside from the interference by the reward-signaling distractor alone (but see Experiment 2 in Pearson et al., 2015). We clearly demonstrated that this reward-induced form of attentional capture is, at least partially, driven by the learning of the stimulus-reward association causing capture to emerge through iterative exposure and is not the consequence or dependent upon salience-mediated capture. Importantly, this is the first study that disentangled effects of explicit knowledge and learning in this form of reward-driven capture, since in previous studies increases in learning were usually confounded with increases in knowledge of the stimulus-reward association.

## Experiment 5: Is explicit knowledge about the reward association necessary for attentional capture by reward-signaling stimuli to occur?

The previous experiment suggests that the reward effect is not instantaneously present but instead needs to be acquired as it gradually emerges over the course of a block. However, like in all our other experiments, participants were explicitly informed about the relationship between the stimuli and the reward they signal. While the results so far suggest that knowledge about the stimulus reward-association is not *sufficient* to characterize the capture by task-irrelevant and non-salient stimuli merely signaling reward (Experiments 3 and 4), it remains unclear whether knowledge about the association is *necessary* to obtain this reward effect. It is feasible that in order to learn the relationship between a feature and the reward availability, the signal needs to be “brought to attention” either by rendering the stimulus physically salient as in Le Pelley et al. (2015) or by informing participants about this relationship (as in the current experiments and those by Failing et al. 2015). In other words, it is possible that without explicit knowledge about the existence of a reliable stimulus-reward association, no learning will take place (cf. Experiment 4) and the reward-signaling stimulus will not interfere with target search. However, if participants learn (i.e., extract information) about the relationship between the feature and the reward it signals even when they are not explicitly informed about it, then one would expect the same effect as in the previous experiments.

Experiment 5 investigated whether participants would learn the relationship between a stimulus and the reward it signals without being explicitly informed about it. For that purpose, participants performed the same task as during the second session of Experiment 1. However, they were only informed that reward could be earned if they would made correct and fast responses and that the amount of reward was not randomly determined. No information was given regarding the relationship between the presence of the particularly colored (red or green) circles and the reward they signaled.

### Materials and methods

#### Participants

Eighteen students from the Vrije Universiteit Amsterdam (15 females, mean age ± 23) participated in Experiment 5. All of the participants had reported normal or corrected-to-normal vision and were naïve as to the purpose of the experiment. None of them had participated in any of the previous experiments. Participants received monetary compensation of between €8 and €14 (€11.00 ± 1.40) based on performance.

#### Apparatus and stimuli

The experimental setup was the same as in the second session of Experiment 1.

#### Procedure and design

The procedure and design were similar to the second session of Experiment 1 (Fig. 1). However, in Experiment 5 participants were only told that they would earn a monetary reward for the correct and fast discrimination of the line segment in the target circle and that the amount of reward was not randomly determined. This information was provided so as to motivate participants to learn about the regularities in the reward payout schedule without providing explicit information about them. No information was given about the relationship between the presence of a particularly colored circle and the reward it signaled. Furthermore, participants were informed that depending on the amount of points they acquired throughout the experiment they could earn up to €14.

In anticipation that it may take longer to learn the feature-reward relationship if not explicitly informed about it, we increased the total number of trials compared to the second session of Experiment 1. Each participant performed 60 trials of practice followed by 10 experimental blocks with the same number of trials yielding a total of 660 trials. Half of the trials in each block featured one specifically colored distractor, and the other half featured the remaining specifically colored distractor.

### Results

To investigate whether a nonsalient and task-irrelevant stimulus signaling relatively high reward affected performance even if participants were not explicitly informed about its reward signal, we ran an ANOVA on mean RT using reward (high vs. low) and block (1-10) as factors. There was no significant main effect of reward, *F*(1,17) = 0.299, *p* = 0.591, but block was significant, *F*(9,153) = 12.603, *p* < 0.001, η^2^ = 0.426. The interaction of reward and block was not reliable *F*(9,153) = 1.222, *p* = 0.303. Participants were numerically equally fast on high reward (494 ms ± 54) compared with low reward trials (494 ms ± 55). Mean RT decreased linearly over blocks, *F*(1,17) = 25.520, *p* < .001, η^2^ = 0.600. There were no significant effects on mean error rate (all *p* > 0.05) which was low in both reward conditions (high vs. low: 6.9% vs. 7.2%).

### Discussion

In Experiment 5, we did not observe interference in search time when a stimulus signaling reward was present. Even though we increased the number of trials to give participants more exposure to the stimulus-reward association compared with previous experiments, no reliable effect emerged.

It is interesting to note that it seems as if the effect did not emerge only because participants were no longer informed about the reward-signaling stimulus. This was in fact the only crucial change in Experiment 5 with respect to the second session of Experiment 1 in which we observed attentional capture by the stimulus signaling relatively high reward. The other difference between the experiments was that in Experiment 5 participants had more exposure to the stimulus-reward association. If anything, this increased exposure should have facilitated the learning of the maladaptive behavior we observed previously.

Compared with the second session of Experiment 1 in which a robust reward effect was found, the results of Experiment 5 suggest that one needs explicit knowledge about the stimulus-reward association for capture by a reward-signaling but otherwise task-irrelevant and nonsalient stimulus to emerge. It might be that knowledge is critically involved in triggering learning of the stimulus-reward association, eventually causing it to capture attention. Because participants were not informed about the association, it is likely that the reward-signaling stimulus never received attentional priority. Without attentional prioritization, learning did not occur which is consistent with other classic studies pointing at the importance of attention in learning (Logan, 1988; Nissen & Bullemer, 1987). This notion will be further explored in the next experiment.

## Experiment 6: On the role of awareness and initial prioritization in attentional capture by reward-signaling stimuli

The absence of an interference effect in search due to the distractor signaling relatively high reward during Experiment 5 might suggest that no capture occurs when participants are not informed about the relationship between the stimulus and the reward it signals. That is, even though participants were more frequently exposed to the reward-signaling stimuli than, for instance, in the second session of Experiment 1, no learning of the stimulus-reward association occurred. Because no learning occurred, no capture by the reward-signaling stimulus was observed.

Alternatively, it may be that, even with extended training, the earlier found reward effect could not be replicated because without explicit knowledge about the association, extracting the reward signal from the heterogeneous display was simply much more sluggish within a given trial. Indeed, the stimulus signaling reward did not stand out from the other non-target elements (at least not due to physical salience as it was the case in Le Pelley et al., 2015) and it was not a task-relevant item as in other studies (Anderson et al., 2011). Similarly, participants in Experiment 5 were not informed about the reward-predictive relationship of the stimulus as in our previous experiments. In other words, because the reward-signaling stimulus did not stand out from the rest of the items in any way it may not have received much, if any, attentional processing making it impossible to establish the association.

With Experiment 6, we aimed to facilitate the extraction of the reward signal by reducing, potentially distracting, task-irrelevant information while maintaining the task-irrelevant and non-salient property of the reward-signaling stimulus. For that reason, we reduced the number of colored elements to two, one of which was the reward-signaling stimulus. Our reasoning was that with only two colored elements the likelihood that participants would process color information would be much greater, allowing them to learn about the association between stimulus and reward.

**Fig. 7:**
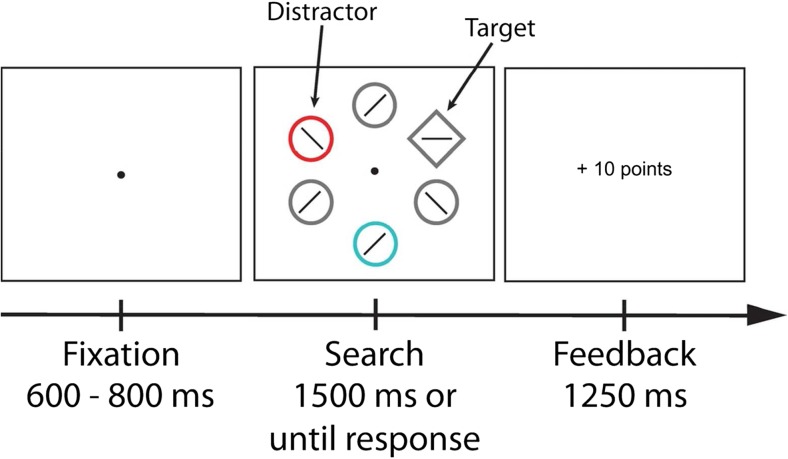
Trial sequence of Experiment 6. Participants were instructed to search for the shape singleton (target; e.g. diamond among circles) and report the orientation of a line segment within the target. The presence of a particularly colored shape (distractor; e.g., red circle) signaled that a high reward could be earned, whereas the presence of a differently colored shape (e.g., green circle) signaled a low reward. The remaining colored shape was of a randomly determined color bearing no consistent reward-predictive signal (here, cyan circle). Participants were not informed about the color-reward relationship. Note that the ISI of 250 ms between the search and feedback display is not shown

In general, the paradigm of Experiment 6 was similar to that of Le Pelley et al. (2015): participants searched for a shape singleton (e.g., a diamond among circles) and responded to the orientation of a line segment inside of it. However, in contrast to Le Pelley et al., all nontarget shapes were gray except one stimulus that had a color signaling the reward availability and one stimulus that had another, randomly chosen, color (Fig. 7). In other words, two shapes in the search display of Experiment 6 were of a particular color (e.g., red and yellow), one of these being the reward-signaling stimulus whose color reliably predicted the magnitude of reward that could be earned. The other colored nontarget was added to ensure that there was no consistent color singleton pop-out of the reward-signaling distractor as was the case in Le Pelley et al.

As in Experiment 5, participants in Experiment 6 were not informed about the stimulus-reward relationship. However, given the more homogenous search display, we anticipated that some participants would become aware of (i.e., “know”) the association. To assess awareness of the stimulus-reward association, each participant performed a final awareness test at the end of the experiment. So far, we observed capture only when participants were informed about the stimulus-reward relationship, which led us to expect that awareness of the association would moderate whether participants show capture by stimuli signaling the availability of relatively high reward. In other words, we expected that participants who are demonstrably aware of the stimulus-reward associations in the final awareness test would show capture by a stimulus signaling relatively high reward while those participants who did not demonstrate awareness of the associations would show no capture during the search task.

### Materials and methods

#### Participants

Thirty-six students from the Vrije Universiteit Amsterdam (25 females, mean age ± 24) with reported normal or corrected-to-normal vision participated in Experiment 6. All participants were naïve as to the purpose of the experiment and had not participated in any of the previous experiments. Participants received monetary compensation of between €8 and €14 (€9.84 ± 0.96) based on their performance.

#### Apparatus and stimuli

Experiment 6 consisted of a visual search task and an awareness test. The experimental setup of the search task was similar to Experiment 2 with a few exceptions. In the experimental task of Experiment 6, four outline shapes including the shape singleton (target) were gray. The remaining two non-target shapes were colored. One of these non-target shapes was of either one of two colors (red or blue), whereas the color of the remaining colored nontarget shape was of a randomly selected color (drawn from green, yellow, pink, brown, or cyan). There was a line segment (1.8°) placed within each shape that could have either one of four different orientations depending on whether it was inside the target shape (0° or 90° angular degrees tilted from vertical) or not (45° or 135°; Fig. 7).

In the awareness test, four awareness displays were successively shown. Each awareness display contained both outline shapes from the visual search task (one circle and one diamond). Both shapes had the same color and were presented next to each other along the horizontal meridian. The color of the shapes was randomly sampled from a pool of four colors (red, blue, green and yellow) without replacement. This pool always contained the two reward-signaling colors (red and blue) as well as two colors that had not reliably predicted reward magnitude (green and yellow).

#### Procedure and design

In Experiment 6, participants first performed the visual search task and then the awareness test. The procedure and design of the visual search task were similar to Experiment 2 with a few exceptions. Participants were instructed to indicate the orientation of a line segment placed inside the target (shape singleton) by pressing the appropriate key (‘X’ for 0° angular degrees tilted from vertical; ‘M’ for 90° tilted; Fig. 7). Participants were merely informed that they would earn a monetary reward for correct and fast discrimination of the line segment in the target circle and that the amount of reward would not be randomly determined. That is, no information was given about the relationship between the presence of a particularly colored shape and the reward it signaled.

Similar to our previous experiments, when participants responded slower than the variable RT limit, they were still able to indicate their response until the trials timed out but would no longer receive any reward for a correct response. However, different to the previous experiments, the variable RT limit of Experiment 6 was based on the 95^th^ percentile of all individual RTs in the preceding block in order to give participants more frequent exposure to the stimulus-reward association. We expected prior to running the experiment that such a liberal RT limit to earn reward would not keep participants motivated to continue improving their RT throughout the entire experiment. To maintain increasingly fast RTs throughout the entire experiment, the variable RT limit for earning reward was based on the 75^th^ percentile during the second half of the experiment. Participants were furthermore informed that depending on the number of points they acquired throughout the experiment they could earn up to €14.

Each participant performed one block of practice followed by 10 experimental blocks of 60 trials each, yielding a total of 660 trials. Half of the trials in each block featured a red and the other half featured a blue nontarget shape in addition to the other randomly colored nontarget shape. Likewise, half of the trials in each block featured a diamond and the other half featured a circle as target shape.

After performing the visual search task, participants were told that the magnitude of reward they could have earned on a given trial was signaled by the presence of a particularly colored nontarget shape. However, they were not informed which color indicated which reward magnitude. Participants were then required to do a final awareness test. In this test, four awareness displays were shown in random order, each containing the two possible outline shapes of the search task (i.e., one circle and one diamond) of either one of four different colors. Participants were informed that of the four colors, one signaled high reward, one signaled low reward, and two colors did not consistently signal any reward. Participants were subsequently asked to indicate which color signaled which reward throughout the task. This part of the awareness test will henceforth be referred to as performed awareness test.

In a final forced-choice task, participants had to indicate whether they considered themselves to be aware of the color that signaled high reward or whether they had guessed which color signaled high reward in the performed awareness test. We refer to this part of the awareness test as reported awareness test.

### Results

To determine whether participants were aware of the stimuli that signaled reward, we first assessed the results of the awareness test. In the reported awareness test, 23 of the 36 participants reported that they were aware of at least the high reward-signaling stimulus throughout the experiment. Twenty-one of these participants correctly identified the color that signaled high reward availability in the performed awareness test. Of the 13 participants who reported to have guessed which color signaled the high reward, only 2 correctly identified the colored stimulus signaling high reward availability in the performed awareness test.

To investigate whether awareness regarding the stimulus-reward relationship differentially affected whether the non-salient and task-irrelevant stimulus signaling relatively high reward affected search performance in Experiment 6, we assigned participants to different groups. Group assignment depended on the results from the final awareness test, separately analyzed for the reported and performed awareness test. For the first group factor, reported awareness, we assigned participants based on their response in the reported awareness test in which they answered the forced-choice question of whether they would consider themselves to be aware of the color that signaled high reward. For the second group factor, performed awareness, we assigned participants based on whether they correctly identified the color that signaled high reward in the performed awareness test.

**Fig. 8:**
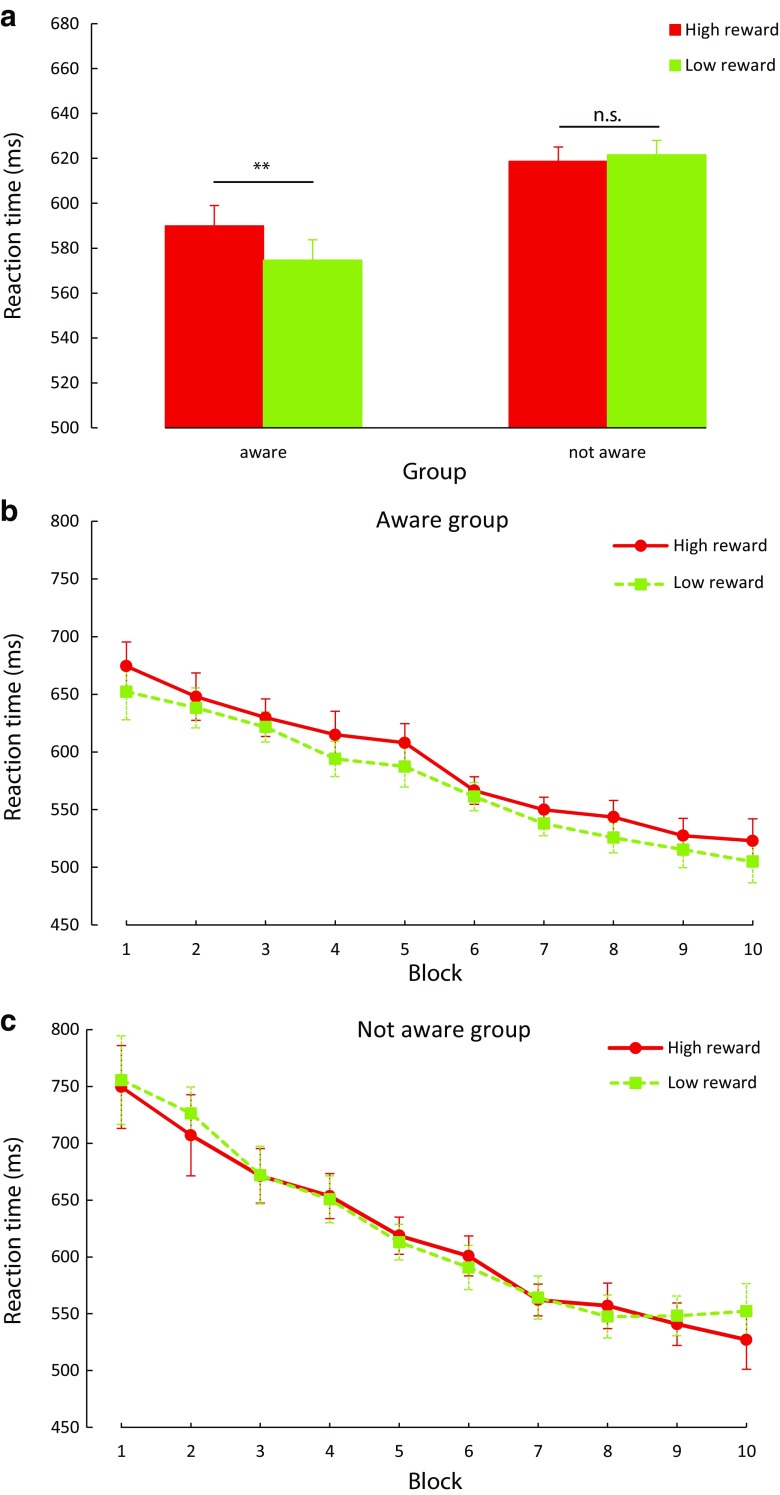
Results of Experiment 6. (**a**) Mean reaction time by reward condition over awareness groups. (**b**) Mean reaction time by reward condition over all blocks of the aware group. (**c**) Mean reaction time by reward condition over all blocks of the not-aware group. For the aware group, mean reaction time was significantly slower in high- compared with low-reward trials. For the not-aware group, there was no significant difference between high- and low-reward trials

To assess whether awareness of the stimulus-reward relationship influenced whether the reward-signaling stimulus in the search display caused interference, we submitted the data to a mixed-design ANOVA with a within-subject factor of reward (high vs. low) and between-subject factor of reported awareness (aware vs. not aware). There was a marginally significant main effect of reward, *F*(1,34) = 3.791, *p* = 0.060, η^2^ = 0.100, and no main effect of reported awareness, *F*(1,34) = 1.865, *p* = 0.181. Crucially, however, there was a significant interaction between reward and reported awareness, *F*(1,34) = 8.125, *p* = 0.007, η^2^ = 0.193. Participants who reported to be aware of the stimulus-reward relationship were significantly slower in high (590 ms ± 87) relative to low reward trials (575 ms ± 81; Fig. 8a), *t*(22) = 3.420, *p* = 0.002, 95% CI [6.03, 24.63], replicating the earlier found reward capture effect. Conversely however, for participants who reported to have guessed which colors signaled which reward, there was no statistically significant difference between high (619 ms ± 72) and low reward trials (622 ms ± 73), *t*(12) = 0.976, *p* = 0.348, 95% CI [−3.55, 9.33]. Similarly, participants who reported to be aware of the stimulus-reward relationship missed out significantly more often on reward payout in the high compared to the low reward condition, *t*(22) = 2.388, *p* = 0.026, 95% CI [1.01, 14.38]. No such difference was found for participants who reported to have guessed which colors signaled which reward, *t*(12) = 0.552, *p* = 0.591, 95% CI [−5.89, 9.89].

A similar analysis on error rates showed a main effect of reward, *F*(1,34) = 4.875, *p* = 0.034, η^2^ = 0.125, no main effect of reported awareness, *F*(1,34) = 2.211, *p* = 0.146, and no interaction, *F*(1,34) = 0.018, *p* = 0.893. On average, participants made slightly more errors in high (10.6%) relative to low reward trials (9.7%), indicating that the difference in RT due to the reward manipulation cannot be the consequence of a speed-accuracy trade-off. Note that the same analyses on mean RT and error rate were performed using performed awareness as between-subject factor. The findings from these analyses were virtually identical to those of the analyses using reported awareness as between-subject factor (see Supplements).

### Discussion

In Experiment 6, we once again observed slower search times on high reward compared to low reward trials. However, this difference was only observed in participants who were aware of the relationship between the stimulus and the reward it signaled. In contrast, participants who were not aware of the stimulus-reward associations showed no statistically significant difference in search times when comparing both reward conditions. For participants who were aware of the stimulus-reward associations, we replicated our previous findings demonstrating that a task-irrelevant and nonsalient stimulus signaling relatively high reward causes attentional capture. More importantly this finding highlights the crucial role of awareness for attentional capture by a task-irrelevant and nonsalient stimulus signaling reward.

The finding that participants who were aware of the stimulus-reward association showed capture even though they were not explicitly informed about the existence or identity of that association illustrates another important point. In all previous experiments, reward-signaling stimuli only captured attention if participants were explicitly instructed about the existence and identity of stimuli signaling reward availability. As such, it may be argued that task instructions are a necessary condition for capture to occur. Experiment 6, however, shows that capture by reward-signaling stimuli is not necessarily triggered by task instructions. Instead, we obtained a reward effect even though no task instructions about the existence of a stimulus-reward association was given. This indicates that participants could not help but to attend the stimulus signaling relatively high reward once they had become aware of the association.

## General discussion

In the current series of experiments, we provide evidence for different conditions under which a reward-signaling stimulus is either beneficial or detrimental to task performance. Most importantly, we demonstrate that stimuli merely signaling reward interfere with target selection, although they were never task-relevant or necessary to obtain reward in any of the present experiments. In fact, attending those stimuli was detrimental, because it would reduce the likelihood to obtain reward due to limited time available to search for the target—a circumstance that participants were explicitly informed about. Crucially, these stimuli were not more physically salient than any other stimulus in the display and therefore would interfere with target selection due to any property other than their reward signal. The findings of the present experiments highlight several important factors concerning the mechanism of how and why task-irrelevant and physically nonsalient stimuli that merely signal reward affect search performance.

In line with previous findings, we show that reward can work as an incentive cue to enhance performance in a visual (search) task. Specifically, we show in Experiment 1 (first session) and Experiment 3 that a cue which signals the magnitude of reward that can be earned on a given trial and which is presented before the onset of the search display “boosts” performance: participants were faster to find the target when they were informed that a relatively higher reward could be obtained on a given trial. This is consistent with studies that show that performance on visual tasks improves as a function of incentive value (Bucker & Theeuwes, 2014; Engelmann & Pessoa, 2007, Sawaki et al., 2015). It is likely that a reward-signaling stimulus presented before the onset of the search display affects task-specific motivation, actively fostering cognitive control when a relatively high reward can be earned (Botvinick & Braver, 2015). Previous studies have suggested that fostering cognitive control due to reward incentives may occur through optimal preparation for the upcoming task (Etzel, Cole, Zacks, Kay, & Braver, 2015; Sawaki et al., 2015). Such optimal preparation is characterized by enhanced efficiency of task encoding and maintenance and may be achieved by means of a stronger task-set representation in frontoparietal regions of the brain (Pessoa & Engelmann, 2010), which are known to play a vital role in (re-)orienting of top-down attention (Corbetta, Patel & Shulman, 2008). Consistent with this notion, recent research demonstrated that incentive cues modulate neuronal activity in these regions (Etzel et al., 2015; Wisniewski, Reverben, Momennejad, Kahnt, Haynes, 2015).

Importantly, we have shown throughout several experiments (Experiment 1, 2, 4 and 6) that when the information about reward availability is presented as a distractor signal *within* the search display, the effect of reward on performance reverses such that participants are slower to respond when a stimulus signaling high reward compared with when a stimulus signaling low reward is present. This reversal in the pattern of search performance, we argue, is due to attentional capture by the stimulus signaling relatively high reward causing significant interference in finding the target. Importantly, the reward-signaling stimulus captured attention even though (1) it was never part of the task set and (2) it was nonsalient, i.e., it was physically equally as salient as any of the other elements in the display. As noted, if anything, selecting the stimulus that signaled reward was detrimental, because it would reduce the likelihood of obtaining reward due to limited time available to search for the target. Despite that, the experiments show that participants were not able to prevent the selection of this stimulus or to develop a strategy to avoid this maladaptive form of attentional capture.

Our experiments demonstrate that maladaptive capture by a reward-signaling stimulus does not occur when reward information is available through other sources, such as through the tone signaling the value before display onset in Experiments 1 and 3. In these experiments, the reward signal presented before the trial functioned as an incentive cue as evidenced by faster search times for high- versus low-reward trials. We speculate that this reflects increased cognitive control allowing for more efficient suppression of distractors while facilitating target selection. Importantly, these findings support the idea that a reward-signaling feature receives priority for attentional selection only if it is, at the time of onset, uniquely predictive of the reward that can be earned on a given trial (Sali et al., 2014).

It should be noted that the type of capture by stimuli that merely signal the availability of reward, as reported here, is quite different from earlier reported forms of reward-driven attentional (Anderson, 2013; Chelazzi et al., 2013; Failing & Theeuwes, 2014) or oculomotor capture (Anderson & Yantis, 2012; Theeuwes & Belopolsky, 2012). These earlier studies provide evidence for a form of capture in which reward is used to promote, and train, an attentional or oculomotor selection response that persists even when reward is omitted in a subsequent experimental phase (cf. instrumental conditioning). Although important, it remained elusive from these studies to what extent task-relevance or physical salience during the initial training phase is critical to observe capture. Similarly, it was unclear whether such, sometimes quite extensive, training was actually necessary to obtain a comparable capture effect when associating reward with a task-irrelevant and non-salient stimulus. In an attempt to discard these concerns, Le Pelley et al. (2015; see also Pearson et al., 2015) showed that reward-signaling stimuli cause capture of attention and the eyes even when these stimuli are always task-irrelevant and no separate training phase is used. However, their study confounded reward with physical salience which limited the conclusion to (physical) salience-driven capture being modulated by reward. Our experiments, however, clearly demonstrated different scenarios in which stimuli that merely signal the magnitude of reward payout do or do not interfere with search performance even if these stimuli are otherwise nonsalient, task-irrelevant and their selection is and was never actively trained.

The increase in search time due to the presence of the reward-signaling distractor could be, in principle, nonspatial in origin and not necessarily be related to spatial attentional capture. According to this notion, distractor interference reflects some form of filtering cost (Kahneman, Treisman, & Burkell, 1983; Treisman, Kahneman, Burkell, 1983) delaying attentional shifts toward the target without any shifts of spatial attention toward the distractor. In other words, a stimulus signaling high reward may induce (larger) nonspatial filtering costs, i.e., a (longer) delay until shifting attention to the target, compared with when such a stimulus signals low reward. While this is feasible, this is unlikely for several reasons. First, a similar reasoning has been used to explain interference in target selection due to the presence of physically salient distractors, especially in the context of the here used paradigm or its numerous variants (i.e., additional singleton paradigm, Theeuwes, 1992; Folk & Remington, 2008; Folk, Remington & Wu, 2009). However, covert attention studies have shown that filtering costs are an unlikely explanation for interference by physically salient distractors in this type of paradigm (Schreij, Theeuwes, & Olivers, 2010). Additionally, previous studies using response interference (Theeuwes, Atchley, & Kramer, 2000), inhibition of return (Theeuwes & Godijn, 2002), or eye movements (Theeuwes et al., 1998, 1999) have clearly established that distractor interference as typically observed in the additional singleton task is due to shifts of spatial attention. Second, research on the influence of reward on attention has demonstrated a strong spatial specificity. For example, Failing and Theeuwes (2014) explicitly tested whether reward-associated stimuli capture attention. They showed both costs and benefits in a cueing paradigm, providing a clear demonstration that reward-associated stimuli cause spatial shifts of attention (see also Peck, Jangraw, Suzuki, Efem, & Gottlieb, 2009; Pool, Brosch, Delplanque, & Sander, 2014). Other studies also observed spatial specificity in many different paradigms (including variants of the additional singleton paradigm) demonstrating spatially lateralized brain responses (Anderson, Laurent, Yantis, 2014; Hickey et al., 2010) as well as oculomotor capture (Anderson & Yantis, 2012; Le Pelley et al., 2015; Theeuwes & Belopolsky, 2012) for reward-associated stimuli. Finally, even when using paradigms and manipulations that are very similar to those used in the present study, oculomotor capture due to reward-signaling stimuli is observed (Failing et al., 2015; Pearson et al., 2016). In summary, we believe that the interpretation in terms of spatial attentional capture, rather than nonspatial interference such as filtering costs, is the most parsimonious and plausible interpretation for the interference by the reward-signaling stimuli as observed here.

Our findings support the notion of learning in this form of attentional capture as initially postulated by Le Pelley et al. (2015; see also Mine & Saiki, 2015). Surprisingly, Le Pelley et al. found very rapidly occurring attentional capture due to reward-signaling stimuli, already evident after relatively few trials, that also did not change over the course of their experiments. As such, they could not provide clear evidence for learning of the stimulus-reward association aside from the interference by the reward-signaling distractor alone. Consistent with these findings, we observed capture to occur rather rapidly (see time course of the second session Experiments 1, 2, and 6 in Figs. 2c, 4, and 8b respectively). However, our Experiment 4, in which we continually swapped the relationship between the reward and the color feature signaling it, provides more detailed evidence for learning the association. Attentional capture in this experiment was not rapidly modulated due to the instructed changes in the color-reward relationship from block to block. Instead, it gradually emerged over iterative exposure to the stimulus and the reward feedback. This supports the notion that learning of the association is a necessary process for the reward-driven capture effect to occur even in the absence of physical saliency promoting attentional capture irrespective of reward availability.

While our experiments provide strong evidence for classical (Pavlovian) conditioning, they also highlight limitations as to how a reward signal affects visual search. According to the notion of classical conditioning, stimuli signaling reward should capture attention merely by virtue of their reward signal, i.e., their Pavlovian association (Le Pelley et al., 2015). Consistent with the notion of a Pavlovian response, we found evidence for learning of capture by a task-irrelevant and nonsalient stimulus signaling reward (Experiment 4). Learning of such a maladaptive behavioral pattern would indeed be predicted by a classical conditioning process since that stimulus had no instrumental but merely signal value (Le Pelley et al., 2016). Clearly, this behavioral pattern cannot be explained in terms of task-relevance or physical salience of the stimulus which could have promoted a response toward the reward-signaling stimulus irrespective of its reward-signaling property. However, observing no capture when participants were not informed or aware of the association between the reward and the stimulus in the search display signaling the reward (Experiments 5 and 6) illustrates an important restriction for when Pavlovian learning affects attentional selection. Indeed, in our experiments, knowledge about the reward relationship seemed to be necessary to observe capture. This finding sets the boundaries for when reward-signaling stimuli capture attention. It suggests that while task-irrelevant and non-salient stimuli merely signaling reward capture attention involuntarily when the stimulus-reward association is learned, triggering learning about the association relies on another mechanism.

A potential explanation for what this mechanism might entail comes from Experiments 5 and 6. On the one hand, our Experiment 6 clearly shows that the fact that we informed participants about the stimulus-reward associations cannot explain the capture we observed previously (second session Experiment 1, 2 and 4). As in Experiment 5, participants were not informed about the stimulus-reward associations. Nevertheless, there was capture in a large subpopulation of the participants. While participants who were aware of the association showed capture by a high reward signaling stimulus, those that were not aware of the stimulus-reward associations did not. This discrepancy underscores that the capture we observed in the previous experiments was not merely triggered by our task instructions. On the other hand, and more importantly however, explicit knowledge about the stimulus-reward association did play an important role. Indeed, our experiments show that only if participants were either explicitly informed (Experiment 1, 2, 4) or became aware of the stimulus-reward association themselves (Experiment 6 as indicated by the final awareness test), attentional capture by stimuli signaling reward is observed. Given these and previous findings, it seems feasible to assume that only once a reward-signaling stimulus is, in some form, prioritized for attentional selection, this stimulus continues to capture attention, independent of other processes known to affect attentional selection.

The claim that attentional prioritization may be necessary for a reward-signaling stimulus to affect search performance is consistent with the long standing acknowledgement in animal conditioning research that attention and learning interact (Mackintosh, 1975; Pearce & Hall, 1980). Conditioning models suggest that stimuli are prioritized for behavior due to their predictive validity. The predictive validity, or predictiveness, describes the ability of a given stimulus (or one of its features) to predict relevant events, such as the administration of a relatively high reward (Le Pelley, 2010; Le Pelley et al., 2015, 2016). It has been argued that an important consequence of a stimulus’ predictiveness is that it “demands” attention (Berridge & Robinson, 1998; Le Pelley et al., 2016). Our experiments clearly support this notion by demonstrating that stimuli that reliably predict upcoming reward capture attention. However, our experiments also demonstrate that the predictive validity of a stimulus alone does not necessarily result in attentional capture. We suggest that—at least in the context of visual search tasks—the process of learning about the reward signal, which eventually becomes a powerful distractor competing with target selection, is only initiated once initial attentional prioritization for that signal is established. Our and previous findings suggest that this is achieved by “guiding” attention to a reward-signaling stimuli either by rendering it task-relevant, physically salient, or, as in the current experiments, by participants themselves becoming aware of the reward signal. It should be emphasized that we suggest that initial attentional prioritization only *triggers* the learning of the stimulus-reward associations. Plenty of evidence from previous studies indicates that initial prioritization by any of the above mentioned means cannot explain why stimulus-reward associations *continue* to bias attentional selection (Anderson, 2013; Chelazzi et al., 2013). That is, the learning of these stimulus-reward associations may eventually come to bias attentional selection above and beyond task-relevance, physical salience, or awareness of the association and even if the initially learned association is no longer in place.

The above outlined mechanism of how learning of the stimulus-reward associations occurs is well-illustrated by the present findings. In our experiments, informing participants about the reward association may have caused initial attentional prioritization of the reward signaling stimuli despite it being detrimental for task performance. As a consequence of this prioritization, learning of the association was triggered and the reward-signaling stimulus started to bias attentional selection. However, when participants had no explicit knowledge about the stimulus-reward association like in Experiment 5, the reward-signaling stimulus was not attentionally prioritized and thus did not capture attention. When reverting back to a simpler and more homogenous search display as in Experiment 6 in which the reward signal was far easier to extract, the capture effect reappeared but only in participants that were aware of the reward signal. Those participants, we argue, attentionally prioritized the reward-signaling stimulus more so than participants that were not aware of the reward signal and—as a consequence—learned the maladaptive behavioral pattern and thus exhibited interference in search time.

Particularly in the context of Experiments 5 and 6, it remains an interesting focus for future research to investigate how participants become aware of the association in the first place. One possibility may be that attention is spontaneously biased towards regularities in associative structure of the feature-reward relationship (Turk-Browne, Junge, & Scholl, 2005; Zhao, Al-Aidroos, Turk-Browne, 2013). Such regularities may be easier to extract if there is less competition between stimuli (compare second session of Experiments 1, 5, and 6). If attention happened to be drawn toward the reward signaling stimulus, the association may strengthen. This possibly leads to reportable awareness of the stimulus-reward association in some individuals but not others which in turn may explain why those that were aware (e.g., in our experiment) eventually exhibited attentional capture.

The idea that to learn a stimulus-reward association, stimuli signaling rewards have to be initially prioritized for attentional selection is well embedded in classic research on how attention moderates learning. For instance, Nissen and Bullemer (1987) demonstrated that participants were not able to reproduce a sequence of flashing lights if they were previously distracted by performing another task (e.g., counting the number of tones presented) while being exposed to the sequence. This led the authors to conclude that acquisition of new associations for memory requires attention. Formalized in the influential theory of automatization, attending to a stimulus is sufficient to commit it to memory and to retrieve from memory whatever has been associated with it in the past (p. 493; Logan, 1988). Even though attention is sufficient for memory encoding, the quality and quantity of attention determine the quality of encoding (Logan, 1988; 2002; Medin & Shaffer, 1978; Moors, 2016). It has been argued that stimuli signaling relatively high reward have a higher incentive salience (Hickey et al., 2010; Berridge & Robinson, 1998) or higher predictive validity (Le Pelley, 2004; Le Pelley et al., 2016) and thus may become more quickly encoded which in turn reinforces their attention-demanding properties.

How can we reconcile the claim about initial attentional prioritization of reward-signaling stimuli and the finding that capture only occurs when one is aware of the presence of the reward-signaling stimuli with the findings from Le Pelley and colleagues (Le Pelley et al., 2015, Pearson et al., 2015)? Note that participants in Le Pelley et al. were not informed about the reward association and yet capture was observed in their experiments. In fact, participants who were aware of the association showed attentional capture by the reward-signaling stimuli that was statistically indistinguishable from participants who were not aware of the association. Similarly, Pearson et al. found no correlation between the size of reward-driven attentional capture and participants’ explicit knowledge of the stimulus-reward relationship. Even though this may appear to be contradictory, there may be a very good explanation for the discrepancy. Indeed, it is likely that in these previous studies initial attentional prioritization was driven by the physical salience of the reward-signaling stimulus. The reward-signaling stimulus in their experiments was always the most salient stimulus in the display (e.g., a red shape among gray shapes), and there is ample evidence that such stimuli capture attention and the eyes in a pure exogenous and, under certain circumstances, even unaware way (Theeuwes, 1992; Theeuwes et al., 1999; Yantis & Egeth, 1999). For example, Theeuwes et al. (1998) showed that observers actually fixated an abrupt onset distractor even though they were not aware of the fact that such an abrupt onset was present. Other studies also have shown that capture by physically salient stimuli occurs in the absence of awareness (Mulckhuyse & Theeuwes, 2010; Zhaoping, 2008). Note that this is not to say, that participant in Le Pelley et al. were unaware of the reward-signaling stimulus, but it makes it less surprising that Le Pelley et al. did not observe differences between the individual awareness groups. Initial prioritization of the reward-signaling stimulus in their experiments did simply not depend on awareness since these stimuli were prioritized due to physical salience. In contrast, we did not observe capture by reward-signaling stimuli without awareness because initial attentional prioritization was not due to physical salience in our experiments.

One could argue that the reward-signaling stimuli in our experiments were, in fact, not task-irrelevant. After all, these stimuli heralded the highest profit which is relevant information to the participants. It therefore may be that participants spatially attended the reward-signaling stimuli to “know” what reward could be earned on a given trial. If knowing about the magnitude of reward was indeed part of the top-down set, then distractor interference may not be surprising after all, as participants just did what they were supposed to do. Yet, on the basis of several arguments, we strongly believe that information about reward magnitude was not part of the volitional top-down set. First, in line with common definitions of top-down control, e.g., “Voluntary or top-down attentional control is driven by current perceptual goals. When an individual is searching for a particular object or feature, or searching in a particular location, they can voluntarily direct overt […] or covert attention […] to the task-relevant object, feature, or location.” (Yantis, Anderson, Wampler, & Laurent, 2012, p. 92), the perceptual goal in our experiments was always clearly defined as a particular target while ignoring distractor stimuli. Moreover, participants were told that the only goal was to search as fast and as accurate as possible for the target, thereby ignoring distractors that would potentially slow them down. Second, participants were informed and also experienced that attending the reward-signaling stimuli would not actually help in receiving any reward. Finally, attending the reward-signaling stimulus impeded them in earning reward. Responses toward the target are slowed when attending the reward-signaling stimulus, which, indeed, led to less reward payout. In other words, although it is clear that the reward-signaling stimuli became relevant to the visual system by means of their reward-signaling property, there is little reason to believe that this was related to an active top-down task-set for attending these stimuli.

Observing attentional capture by reward-signaling stimuli after being instructed to ignore these stimuli bears some resemblance to the attentional white bear phenomenon (Lahav, Makovski, & Tsal, 2012; Tsal & Makovski, 2006). According to this notion, participants tend to attend distractors they are instructed to ignore. Even though a feasible account, several observations make it unlikely that this is an explanation for the present findings. For example, there was no capture for the reward-signaling stimuli in Experiment 3 even though participants were instructed to ignore them. Similarly, we observed attentional capture in Experiment 6 even without an explicit instruction to ignore reward-signaling stimuli (i.e., any mention of such stimuli). Finally, it is unclear how the white bear phenomenon can account for the difference in attentional priority between stimuli signaling high or low reward, because participants were instructed to ignore any type of reward-signaling stimulus. Although it is unlikely that the white bear phenomenon can account for the reward effect, it may still be possible that it serves as a prioritizating agent that enables learning about the reward association under certain conditions.

## Conclusions

The current experiments provide evidence for attentional capture by stimuli that merely signal reward even though selecting those stimuli is and was never predictive or necessary for actual reward payout. Deterioration of search performance due to attentional capture by a reward-signaling stimulus is characterized by a learning pattern and can neither be explained in terms of an active task set, task relevance or physical salience of that stimulus. Instead, the present data show that knowledge about or awareness of the stimulus-reward association moderates whether task-irrelevant and non-salient stimuli that merely signal reward capture attention. On a broader scale, our findings suggest that initial prioritization of a reward-signaling stimulus, either by means of task-relevance, physical salience, or awareness of the stimulus-reward association is necessary to trigger the learning process of this maladaptive behavioral pattern. It is only after initial attentional prioritization that a reward-signaling stimulus causes capture in visual search above and beyond top-down and bottom-up signals.

## Electronic supplementary material

Below is the link to the electronic supplementary material.

ESM 1 (DOCX 14 kb)
