# Supplementary material for: Don’t let it distract you: how information about the availability of reward affects attentional selection
Source: Atten Percept Psychophys. 2017 Jul 21;79(8):2275–98. doi: 10.3758/s13414-017-1376-8 (PMC5662709; doi:10.3758/s13414-017-1376-8)
Supplement: Supplementary file 1 — (DOCX 14 kb) [file 13414_2017_1376_MOESM1_ESM.docx]

**Supplements**

The previous analysis indicates a difference in the reward effect for those participants who were aware of the stimulus-reward relationship compared to those who were not. To examine how this reward effect changed over the course of the experiment for either of the reported awareness groups, data from the search task were submitted to a mixed-design ANOVA with reward (high vs. low), block (1^st^-10^th^) and reported awareness (aware vs. not aware) as factors. There was a marginally significant main effect of reward, *F*(1,34) = 3.109, *p* = .087, η² = .070, a main effect of block, *F*(9,306) = 3.109, *p* < .001, η² = .751, and no main effect of reported awareness, *F*(1,34) = 1.955, *p* = .171. Regarding the interactions there was a significant interaction between reward and reported awareness, *F*(1,34) = 7.565, *p* = .009, η² = .169, no interaction between reward and block, *F*(9,306) = 1.297, *p* = .255, a significant interaction between block and reported awareness, *F*(9,306) = 4.387, *p* = .006, η² = .028, and no three-way interaction between reward, block and reported awareness, *F*(9,306) = 1.346, *p* = .232. While search RT decreased in both reported awareness groups over the course of the experiment, participants who did not report awareness of the stimulus-reward relationship showed a greater overall reduction, *F*(1,34) = 6.021, *p* = .019, η² = .150. As Figure 8b and 8c illustrate, this most likely stems from a slower overall mean RT in the first blocks. At around the fifth block, all participants converged onto similar average search times and a comparable slope. It is evident, however, that the reward effect did not change significantly over blocks for either of the two reported awareness groups.

A similar analysis on error rates showed a main effect of reward, *F*(1,34) = 4.875, *p* = .034, η² = .125, block, *F*(9,306) = 5.288, *p* = .001, η² = .135, and no main effect of reported awareness, *F*(1,34) = 2.211, *p* = .146. None of the interactions was statistically significant (all *p* > .05), suggesting that error rates remained relatively stable over the course of the experiment.
